# Targeting stromal remodeling and cancer stem cell plasticity overcomes chemoresistance in triple negative breast cancer

**DOI:** 10.1038/s41467-018-05220-6

**Published:** 2018-07-24

**Authors:** Aurélie S. Cazet, Mun N. Hui, Benjamin L. Elsworth, Sunny Z. Wu, Daniel Roden, Chia-Ling Chan, Joanna N. Skhinas, Raphaël Collot, Jessica Yang, Kate Harvey, M. Zahied Johan, Caroline Cooper, Radhika Nair, David Herrmann, Andrea McFarland, Niantao Deng, Manuel Ruiz-Borrego, Federico Rojo, José M. Trigo, Susana Bezares, Rosalía Caballero, Elgene Lim, Paul Timpson, Sandra O’Toole, D. Neil Watkins, Thomas R. Cox, Michael S. Samuel, Miguel Martín, Alexander Swarbrick

**Affiliations:** 1 Garvan Institute of Medical Research, Darlinghurst, NSW 2010 Australia; 2 The Kinghorn Cancer Centre, Darlinghurst, NSW 2010 Australia; 3 St Vincent’s Clinical School, Faculty of Medicine, UNSW Sydney, Darlinghurst, NSW 2010 Australia; 4 The Chris O’ Brien Lifehouse, Camperdown, NSW 2050 Australia; 5 Royal Prince Alfred Hospital, Camperdown, NSW 2050 Australia; 6 MRC Integrative Epidemiology Unit, University of Bristol, Oakfield House, Bristol, BS8 2BN UK; 7 Centre for Cancer Biology, SA Pathology and the University of South Australia, Adelaide, SA 5000 Australia; 8 Faculty of Health Sciences, School of Medicine, University of Adelaide, Adelaide, SA 5000 Australia; 9 Pathology Queensland and School of Medicine, University of Queensland, St Lucia, QLD 4006 Australia; 10 Rajiv Gandhi Centre for Biotechnology, Thycaud Post, Poojappura, Thiruvananthapuram, Kerala 695014 India; 11 Department of Medical Oncology, Hospital Universitario Virgen del Rocío, 41013 Sevilla, Spain; 12 Department of Pathology, Hospital Universitario Fundación Jiménez Díaz, 28040 Madrid, Spain; 13 Department of Medical Oncology, Hospital Clínico Universitario Virgen de la Victoria, IBIMA, 29010 Málaga, Spain; 14 GEICAM, Spanish Breast Cancer Group, Madrid, 28703 Spain; 15 St Vincent’s Hospital, 2010 Darlinghurst, NSW Australia; 16 Department of Medical Oncology, Instituto de Investigación Sanitaria Gregorio Marañón, Universidad Complutense, Centro de Investigación Biomédica en Red de Oncología, CIBERONC-ISCIII, 28040 Madrid, Spain

## Abstract

The cellular and molecular basis of stromal cell recruitment, activation and crosstalk in carcinomas is poorly understood, limiting the development of targeted anti-stromal therapies. In mouse models of triple negative breast cancer (TNBC), Hedgehog ligand produced by neoplastic cells reprograms cancer-associated fibroblasts (CAFs) to provide a supportive niche for the acquisition of a chemo-resistant, cancer stem cell (CSC) phenotype via FGF5 expression and production of fibrillar collagen. Stromal treatment of patient-derived xenografts with smoothened inhibitors (SMOi) downregulates CSC markers expression and sensitizes tumors to docetaxel, leading to markedly improved survival and reduced metastatic burden. In the phase I clinical trial EDALINE, 3 of 12 patients with metastatic TNBC derived clinical benefit from combination therapy with the SMOi Sonidegib and docetaxel chemotherapy, with one patient experiencing a complete response. These studies identify Hedgehog signaling to CAFs as a novel mediator of CSC plasticity and an exciting new therapeutic target in TNBC.

## Abstract (web summary)

Stromal cell recruitment, activation and crosstalk with cancer cells is poorly understood. Here, the authors demonstrate that cancer cell-derived Hedgehog ligand  triggers stromal remodeling that in turn induces a cancer-stem-cell like, drug-resistant phenotype of nearby cancer cells while treatment with smoothened inhibitors reverses these phenotypes.

## Introduction

Carcinogenesis draws many parallels with developmental biology. During development, dynamic interaction between stromal and epithelial cells drives patterning and function. Cell fate specification occurs through activation of transcriptional cascades in response to extracellular signals from developmental signaling pathways such as Hedgehog (Hh), Wnt, Notch, BMP (bone morphogenetic proteins), and FGF (fibroblast growth factor)^1,2^. These pathways direct developmental processes either by direct cell-to-cell contact or through secreted diffusible factors (paracrine signaling). They can act individually or in concert with each other. For example, the interaction between Hh and FGF signaling pathways has been shown to mediate tracheal and lung branching morphogenesis^3^. In mature, differentiated tissues, these pathways are quiescent but may be reactivated to drive repair and regeneration to maintain tissue homeostasis.

More specifically, the Hh developmental pathway is reactivated in a subset of cancers. Binding of Hh ligand to its receptor Patched (PTCH) enables Smoothened (SMO)-mediated translocation of Gli1 into the cell nucleus to drive the transcription of Hh target genes^4^. Mutations in Hh pathway components are oncogenic drivers in “Gorlin’s-like” cancers such as medulloblastoma and basal cell carcinoma where tumors rely on cell-autonomous Hh signaling^5^. Small molecule inhibitors of SMO (SMOi), Vismodegib and Sonidegib, are well-tolerated and clinically approved for the treatment of these lesions^5^. In contrast, many other solid tumors, including breast cancer, predominantly exhibit ligand-dependent pathway activation^4–6^. While Hh signaling is quiescent in the adult mammary gland, Hh ligand expression is reactivated in a subset of breast cancers, particularly the poor-prognosis triple negative breast cancer (TNBC) subtype^6^. Thirty percent of TNBCs exhibit paracrine Hh pathway signature, defined by high epithelial HH ligand expression in combination with high stromal GLI1 expression, which is associated with a greater risk of metastasis and breast cancer-specific death^6^.

Neoplastic cells co-opt components of the tumor microenvironment (TME) to further their progression. The TME is a complex ecosystem comprising a myriad of neoplastic and nonmalignant cells embedded in a glycoprotein-rich extracellular matrix (ECM). Prominent cell types include the endothelium, cells of the immune system and cancer-associated fibroblasts (CAFs). In addition to its role as a physical scaffold to support tissue architecture, the ECM also functions as a signal transducer between the different TME cell types^7^. The stiffness of the ECM and the abundance of fibrillar collagen immediately adjacent to epithelial lesions provide mechanical signals that facilitate tumor development and progression^8,9^. Not surprisingly, the TME has emerged as a major determinant of cancer phenotype. In breast cancer, stromal metagenes, in particular those associated with ECM remodeling, strongly predict prognosis and response to chemotherapy^10,11^.

While it is now apparent that Hh signals in a paracrine manner in animal models of TNBC^6^ and in isolated cancer stem cells (CSCs)^12^, a detailed study of the dynamic crosstalk within the TME is required to make clinical progress in integrating anti-stromal therapies into breast cancer treatment. Progress has been impeded by the field’s limited understanding of the mechanisms underlying tumor−stromal interactions, a limited repertoire of well-tolerated agents to target the TME, and an absence of predictive biomarkers for response to TME-directed therapies. In this study, we showed that CAFs are the primary stromal cells that respond to Hh ligand stimulation. Activated CAFs in turn provide a conducive environment for neoplastic cells to acquire a chemo-resistant stem-like phenotype. SMOi treatment sensitized tumors to docetaxel chemotherapy in mouse models and in patients from the EDALINE Phase I clinical trial, resulting in reduced metastatic burden and improved survival.

## Results

### Hh-tumors are enriched for a reversible CSC-like phenotype

To investigate the mechanistic basis for Hh-dependent tumor growth and metastasis in TNBC, we used the murine M6 allograft model of low grade TNBC, in which transgenic Hh expression drives invasion, metastasis, and high-grade morphology^6^ (M6-Hh; Supplementary Fig. 1a). Treatment with the SMO inhibitor (SMOi) GDC-0449 (Vismodegib; 100 mg/kg/bid) slowed tumor growth, reduced metastatic burden and improved overall animal survival of M6-Hh tumors (Supplementary Fig. 1b–d). M6-Ctrl and M6-Hh monoculture cell viability were similar between vehicle and SMOi treatment and the expression of canonical Hh target genes *Ptch*, *Gli1*, and *Hhip* were downregulated in vivo but not in vitro, consistent with a paracrine requirement for Hh signaling as previously reported^6,12,13^ (Supplementary Fig. 1e–g). The effects of SMO inhibition on tumor growth and gene expression were not observed in control tumors lacking Hh expression (M6-Ctrl) or in benign adult mouse mammary gland (Supplementary Fig. 1b, g, h), reflecting on-target drug activities. Similar results were observed with the SMOi NVP-LDE-225 (Sonidegib; 80 mg/kg/day; Supplementary Fig. 1i, j).

**Fig. 1:**
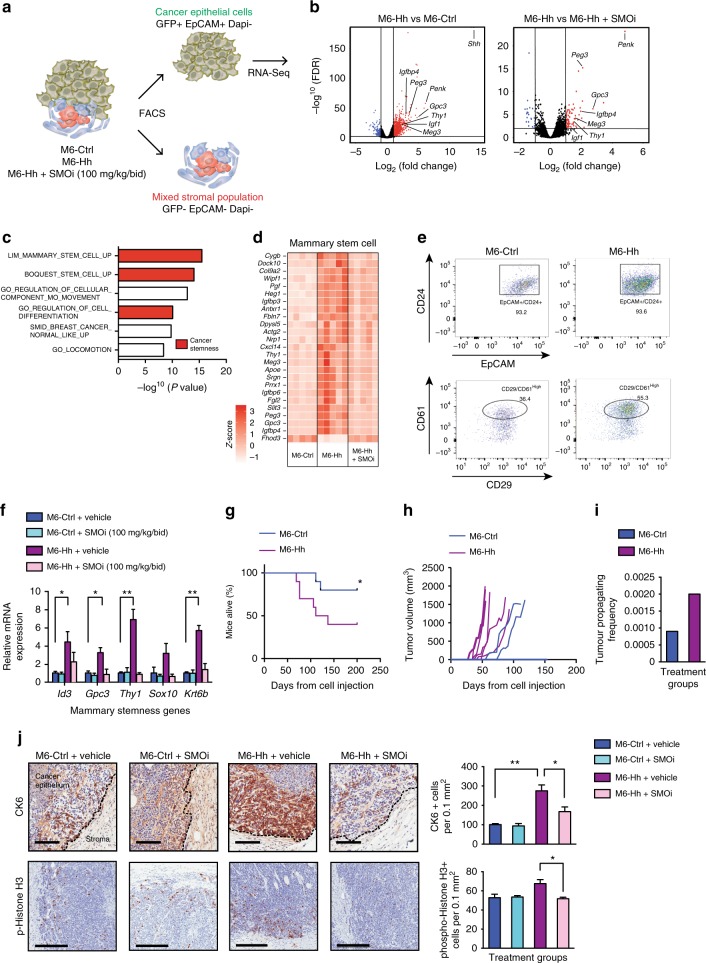
Malignant epithelial cells with increased self-renewal properties are located adjacent to the stroma of Hh-expressing cancers. **a** Scheme depicting the purification of epithelial and mixed stromal populations from disaggregated M6 murine tumor models. **b** Expression of genes significantly downregulated (blue) or upregulated (red) by RNA-Seq analysis (cut-off log_2_ (Fold Change) ≥ 2; vertical lines) in the epithelium of M6-Hh tumors compared to the epithelium of M6-Ctrl or M6-Hh tumors treated with the SMOi, GDC-0449 (100 mg/kg/bid), plotted against FDR values (horizontal lines indicate −log^10^ (FDR) > 2). Each symbol represents the transcriptome from five biological replicates per treatment group. **c** GSEA analysis reveals significant enrichment for genes encoding stemness and invasion in M6-Hh primary cells (FDR *q* value < 0.05). **d** Heat map showing relative CSC genes expression in the epithelium of M6-Hh tumors compared to M6-Ctrl and M6-Hh tumors + SMOi. Data show normalized row *Z*-score (*n* = 5 biological replicates for each treatment group). **e** Representative FACS dot plots showing the expression of CSC markers CD61 and CD29 within the EpCAM^+^/GFP^+^/CD24^+^ population of M6-Ctrl and M6-Hh tumors (*n* = 3 biological replicates per group). **f** Relative expression of key stemness genes in M6-Ctrl and M6-Hh whole tumors ± SMOi (100 mg/kg/bid). *n* = 3 biological replicates per treatment group; statistical significance was determined using unpaired two-tailed Student’s *t* test with equal s.d. **g** Kaplan−Meier and **h** tumor penetrance curves of mice injected with 250 primary M6-Ctrl (blue) or M6-Hh cells (violet); *n* = 10 biological replicates. Six tumors from the M6-Hh and two tumors from the M6-Ctrl models were detectable, respectively. Statistical significance was determined using log-rank test. **i** Primary M6-Ctrl and M6-Hh tumor cells were isolated by FACS and transplanted at various dilutions into recipient mice. Limiting dilution analysis demonstrating higher in vivo tumor-forming capacity in M6-Hh cells compared to M6-Ctrl cells. *n* = 10 mice per condition. **j** Representative images and quantification of CK6-progenitor and phospho-Histone H3-positive cancer cells at the tumor−stromal interface of M6 tumor models. Scale bars: 100 μm for CK6 and 200 μm for phospho-Histone H3. *n* = 3 biological replicates per treatment group. Statistical significance was determined using unpaired two-tailed Student’s *t* test; *P < 0.05; **P < 0.01. Bars represent mean ± s.e.m.

To examine the transcriptional changes induced by Hh pathway activation in detail, we dissociated and analyzed freshly flow cytometry-sorted stromal and epithelial fractions of M6-Ctrl and M6-Hh tumors ± SMOi using RNA-Sequencing (RNA-Seq) (Fig. 1a). Differential gene expression analysis confirmed effective cellular fractionation, with Hh transgene expression restricted to the epithelial cell population while Hh target genes *Ptch*, *Gli1*, and *Hhip* expression were induced solely in the stromal fraction of M6-Hh tumors (Supplementary Data 1).

In the epithelial compartment, 67 genes were differentially expressed (>2-fold change, *P* < 0.001), with 60 upregulated and 7 downregulated genes in the neoplastic M6-Hh cells in comparison to M6-Ctrl or M6-Hh cells treated with SMOi. Transcriptional changes were robust and highly statistically significant (Fig. 1b). Hh expression in M6 cancer cells resulted in increased expression of stemness genes^14^ including *Peg3* (>11-fold), *Igfbp4* (>7-fold), and *Thy1* (>4-fold), which were downregulated following SMO inhibition (Fig. 1b).

Gene set enrichment analysis (GSEA) and gene ontology (GO) analysis of the purified epithelial fraction highlighted enrichment for genes specifically and almost exclusively associated with mammary stemness and invasion, consistent with the morphologically undifferentiated phenotype previously observed in Hh-overexpressing tumors^6^ (Fig. 1c, d and Supplementary Data 1). To examine the CSC phenotype in greater detail, epithelial cells were profiled by flow cytometry. M6-Hh tumors had a higher proportion of CD61^hi^ cells, previously shown to be a marker of mouse mammary CSCs^15^, within the EpCAM^+^CD24^hi^CD29^+^ cancer cell population (55.3% in M6-Hh tumors vs. 36.4% in M6-Ctrl  tumors; Fig. 1e). M6-Hh tumors also had elevated expression of the stemness markers *Id3*, *Gpc3*, *Thy1*, *Sox10*, *and Krt6*, validating the RNA-Seq data (Fig. 1b, f). Following transplantation of low numbers of sorted primary M6-Hh and M6-Ctrl cells into naive recipients, tumors were detectable earlier, formed at a higher frequency and survival was shorter in the M6-Hh group (Fig. 1g, h). Limiting dilution assays^16^ were used to quantitate the impact of Hh signaling on tumor-initiating capacity. M6-Ctrl and M6-Hh tumor cells were isolated by FACS and transplanted at various dilutions. M6-Hh cells had significantly higher tumor-initiating capacity (1 in 435) compared with M6-Ctrl cells (1 in 1088; Fig. 1i). Importantly, the proliferation and expression of CSC markers were indistinguishable between M6-Ctrl and M6-Hh cells in monoculture, indicating that Hh expression in M6 cells does not regulate CSC properties in a cell-autonomous manner (Supplementary Fig. 1e).

Immunohistochemical detection of the mammary progenitor marker cytokeratin 6 (CK6; product of the *Krt6* gene)^17^ localized cells with a stem/progenitor signature specifically to the tumor−stromal interface (Fig. 1j). SMO inhibition reduced the expression of *Id3*, *Gpc3*, *Thy1*, *Sox10*, *and Krt6* and significantly reduced the number of cells positive for CK6 and the mitotic marker phospho-Histone H3 at the tumor−stromal interface (Fig. 1f, j). These data demonstrate that paracrine Hh signaling results in the induction of a reversible stem-like phenotype preferentially at the tumor−stromal interface.

### Hh-activated CAFs markedly remodel the ECM

**Fig. 2:**
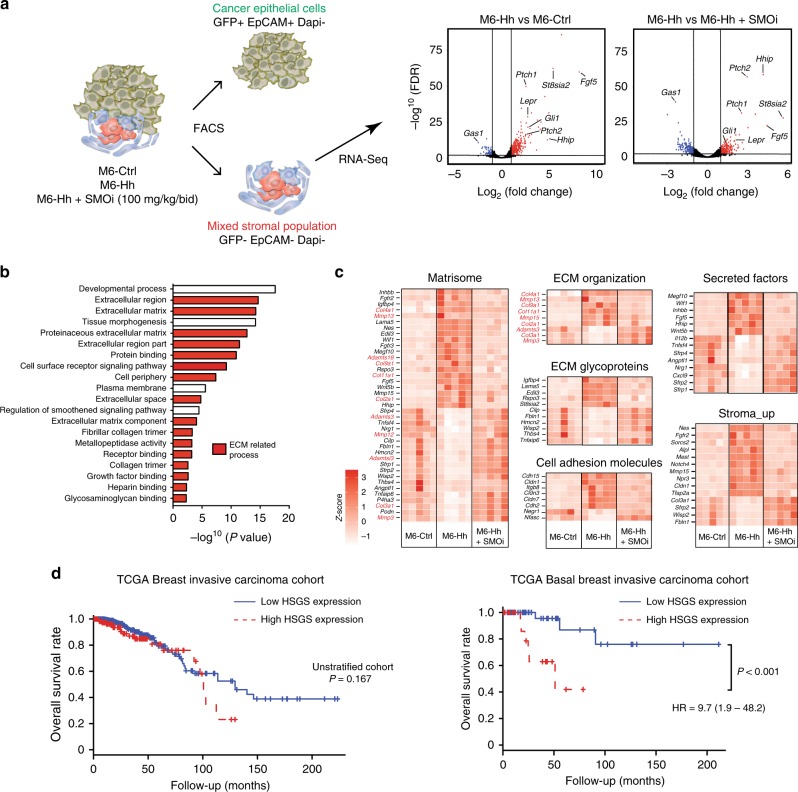
Hh-activated stroma defines a novel transcriptional signature robustly associated with poor-prognosis in TNBC. **a** Expression of genes significantly downregulated (blue) or upregulated (red) (cut-off log_2_ (Fold Change) ≥ 2; vertical lines) in the stroma of M6-Hh tumors compared to the stroma of M6-Ctrl or M6-Hh tumors treated with SMOi (100 mg/kg/bid), plotted against FDR values (horizontal lines indicate −log^10^ (FDR) > 2). Each symbol represents the transcriptome of the stromal population from five biological replicates per treatment group. **b** RNA-Seq analysis reveals significant enrichment of GO groups related to ECM organization and production in M6-Hh tumor stroma (FDR *q* value < 0.05). **c** Heat maps demonstrating relative expression levels of gene-sets defined by RNA-Seq analysis. Genes highlighted in red represent those that are directly involved in ECM production. Data show normalized row *Z*-score (*n* = 5 biological replicates for each treatment group). **d** Kaplan−Meier curves of overall survival in unstratified breast cancer patients and in patients with basal, TNBC. Blue and red lines represent low and high Hh-stromal gene signature expression (HSGS), respectively. Statistical significance was determined using the Log-rank test; ***P < 0.001

RNA-Seq analysis of the stromal fraction revealed 185 genes that were differentially expressed (>2-fold, *P* < 0.001), with 146 upregulated and 39 downregulated genes in the stroma of M6-Hh tumors compared to M6-Ctrl and M6-Hh tumor + SMOi (Supplementary Data 1). A number of genes were markedly upregulated by Hh signaling, in particular the growth factor gene *Fgf5* at more than 290-fold, *St8Sia2* (>40-fold) and *Tspan11* (>4-fold; Fig. 2a). The large majority of gene expression changes in Hh-activated stroma returned to baseline following treatment with SMOi (Fig. 2a and Supplementary Data 1), suggesting that the stromal transcriptional changes are SMO-dependent and reversible.

Comparative GO and GSEA analysis revealed that the stromal fraction of M6-Hh tumors are highly enriched for genes encoding ECM processes (Fig. 2b, c), suggesting that a major influence of paracrine Hh activation on the tumor stroma is related to ECM production and remodeling^18^. Genes highly enriched in this set included collagens (*Col2a1, Col3a1, Col4a1*, *Col9a1*, *Col11a1*), ECM remodeling metallopeptidases (*Mmp3, Mmp13*, *Mmp15*, *Adamts3, Adamts18*), ECM glycoproteins (*St8sia2*, *Rspo3*, *Lama5*, *Edil3*, *Thbs4*) and cell adhesion molecules (*Cldn3*, *Cldn7*, *Cldn1*, *Cdh2*, *Cdh15*) (Fig. 2c). Interestingly, these ECM gene expression changes were not a consequence of changes in stromal cellular composition due to Hh pathway activation or long-term SMO inhibition. Immunohistochemical analysis did not reveal any difference between M6-Ctrl and M6-Hh tumors in terms of CAF, endothelial and innate immune cell abundance, regardless of treatment with SMOi (Supplementary Fig. 2a). This finding was confirmed using whole tumor qRT-PCR and flow cytometry analysis (Supplementary Fig. 2b, c).

To determine the prognostic value of the Hh-activated stromal gene signature (HSGS), we examined its impact on overall survival using The Cancer Genome Atlas (TCGA) breast invasive carcinoma cohort^19^. The HSGS was not predictive of patient outcome in the unstratified patient cohort (Fig. 2d) but was associated with significantly lower patient overall survival uniquely in the basal breast cancer subtype, where Hh ligand is most frequently overexpressed^6^ (Hazard ratio = 9.7 (1.9−48.2); *P* < 0.001; Fig. 2d).

Accumulating evidence suggests that CAFs contribute to tumor growth upon Hh ligand activation^12,20^. However, the stroma of M6 tumors is composed of multiple cell types, any of which may be responsible for Hh-dependent gene expression changes. We used a single-cell RNA-sequencing (scRNA-Seq) approach to determine the cell population/s that respond to paracrine Hh signaling. The microfluidic 10× Chromium system was used to comprehensively profile gene expression at cellular resolution in thousands of cells isolated from freshly dissociated M6-Ctrl and M6-Hh tumors ± SMOi. In total, we compared 6064 FACS-isolated cells from M6-Ctrl tumors, 6200 cells from M6-Hh tumors, and 2686 single cells from M6-Hh tumor treated with SMOi.

**Fig. 3:**
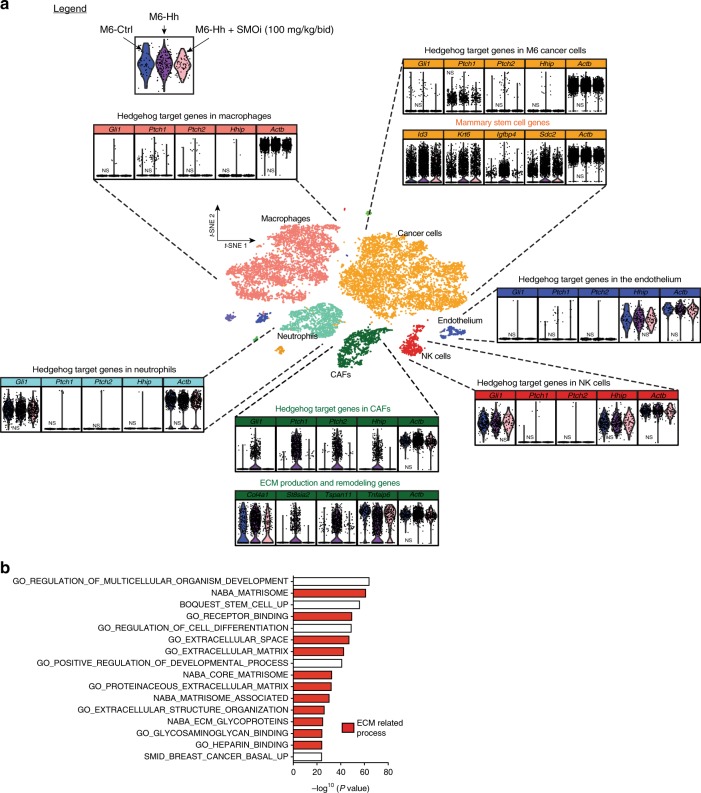
Paracrine Hedgehog signaling at cellular resolution. **a** Freshly isolated M6-Ctrl (blue), M6-Hh (magenta) and M6-Hh tumors + SMOi (100 mg/kg/bid; pink violin plot) were captured using 10x Chromium technology and the Cell Ranger Single Cell Software Suite 2.0 was used to perform demultiplexing, barcode processing, and single-cell 3′gene counting. *t*-SNE plot shows the subcellular clusters present in the breast TME of M6 tumors. Unless stated, *P* values are highly significant (Supplementary Data 2). **b** Single-cell RNA-Seq analysis reveals significant enrichment of GSEA groups related to ECM organization and production specifically in the CAF population of M6-Hh tumors (ECM-related processes highlighted in red; FDR *q* value < 0.05)

As shown in Fig. 3a, unsupervised clustering analysis of 14,950 cells revealed populations of myeloid, neoplastic, endothelial, CAF and natural killer cells within the breast TME (Fig. 3a). Importantly, the upregulation of canonical Hh target genes *Gli1*, *Ptch1*, *Ptch2*, and *Hhip* and ECM genes such as *Col4a1*, *Tspan11*, *St8sia2*, and * Tnfaip6* was observed exclusively in the CAF population of M6-Hh tumors (Fig. 3a), and not in the other stromal cell types. More specifically, the ECM signature detected in the stroma of Hh-expressing tumors via “bulk” RNA-Seq was driven by CAF gene expression (Figs. 2b, c, 3b). scRNA-Seq also confirmed the lack of autocrine Hh pathway activation within the neoplastic cells (Fig. 3a). Treatment with SMOi almost completely reversed the Hh-dependent gene expression changes observed in CAFs without affecting gene expression in the other stromal cell types (Fig. 3a; Supplementary Fig. 2d and Supplementary Data 2), highlighting the on-target activity of SMOi at the single-cell level.

Coculture of primary CAFs with M6-Hh cells was sufficient to induce the expression of the Hh target genes *Ptch2*, *Gli1*, and *Hhip* in CAFs (Supplementary Fig. 3a, b) with concomitant upregulation of CSC markers *Id3*, * Sox10*, *Itgb3*, and *Krt6b* in M6-Hh cells (Supplementary Fig. 3a, c). Importantly, this stromal-epithelial malignant crosstalk was blocked by SMOi (Fig. 3a, Supplementary Fig. 3b, c). These data allow us to conclude that Hh signaling occurs solely in a paracrine manner in this murine model of TNBC and that CAFs are the therapeutic target of SMOi in TNBC.

### Hh-dependent collagen remodeling promotes cancer stemness

**Fig. 4:**
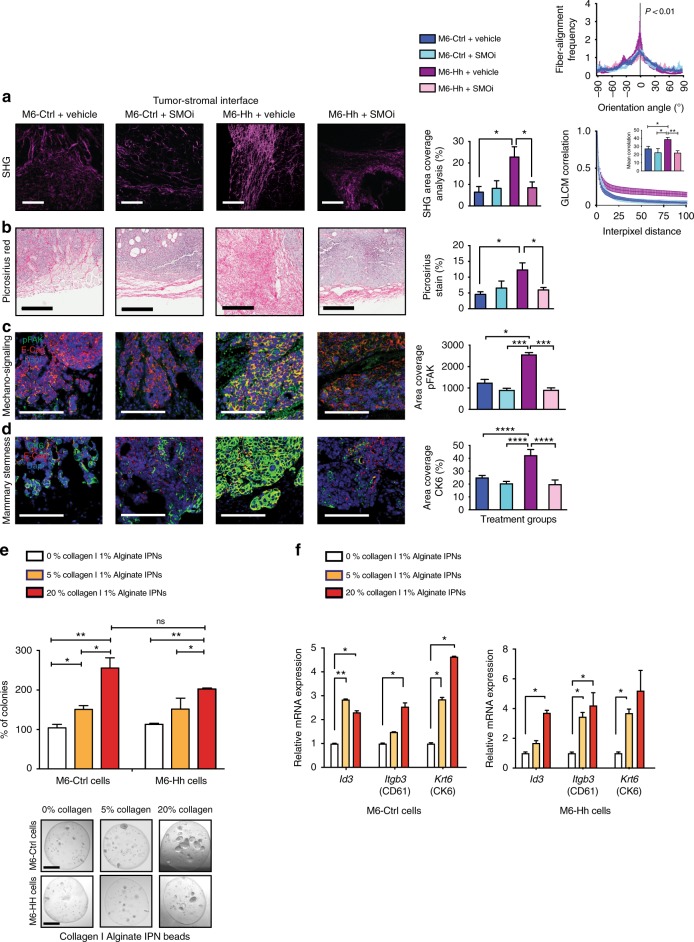
High fibrillar collagen content resulting from Hh pathway activation promotes mechano-signaling and breast cancer stemness. **a**–**d** Concomitant expression analysis of collagen content and organization, integrin/focal adhesion activation and CSC-like characteristics at the tumor−stromal interface of M6 tumor models ± SMOi (100 mg/kg/bid; *n* *=* 3 biological replicates). **a** Representative multiphoton SHG imaging (scale bars: 100 μm) and quantitative analysis of collagen abundance. Statistical significance was determined using unpaired two-tailed Student’s *t* test with equal s.d. Corresponding graphs comparing fiber orientation (top right panel) and quantifying GLCM (bottom right panel). Unpaired two-tailed nonparametric Mann–Whitney *U* test was used for determining statistical significance across distributions. For GLCM analysis, statistical significance was determined using unpaired two-tailed Student’s *t* test with equal s.d. **b** Collagen I and III deposition detected and quantified by picrosirius red staining. Scale bars, 200 μm. Statistical significance was determined using unpaired two-tailed Student’s *t* test with equal s.d. **c** Representative immunofluorescence images and quantification of phospho-FAK and **d** CK6 expression. Scale bars, 100 μm. Statistical significance was determined using Kruskal−Wallis test. **e** M6-Ctrl and M6-Hh single cells were embedded and grown for 12 days in 3D Alginate IPNs containing increasing concentrations of collagen. Quantification of the number of colonies was normalized to the mean colony count in 0% collagen IPNs. *n* = 3 biological replicates with at least six technical replicates per condition. Statistical significance was determined using unpaired two-tailed Student’s *t* test with equal s.d. Representative phase contrast images of M6 colonies on polyacrylamide substrata after 12 days of culture. Scale bars: 100 μm. **f** Relative mRNA expression of the CSC markers *Id3*, *Igtb3*, and *Krt6b* in M6-Ctrl and M6-Hh cells cultured within 3D alginate-collagen I IPNs. *n* = 3 biological replicates with six technical replicates per experiment. Statistical significance was determined using unpaired two-tailed Student’s *t* test with equal s.d. *P < 0.05; **P < 0.01; ***P < 0.001; ****P < 0.0001. Bars represent mean ± s.e.m.

Bulk and scRNA-Seq data suggested that stromal Hh-signaling drives collagen remodeling in the local ECM (Figs. 2b, c,  3), which is known to associate with breast cancer progression^21^. We employed second harmonic generation (SHG) microscopy, a sensitive label-free method for quantifying fibrillar collagen density and orientation in tissues^22^. SHG analysis revealed a ~3-fold increase in fibrillar collagen density at the tumor−stromal interface of Hh-expressing tumors (Fig. 4a), but not in the tumor center (data not shown). The increase in collagen abundance was confirmed by chromogenic staining using Picrosirius red (Fig. 4b). Further detailed analyses of the distribution and orientation of collagen fibers as described by Mayorca-Guiliani et al.^23^ and by Gray level co-occurrence matrix (GLCM) analysis^24^ revealed changes in texture and crosslinking of the ECM with linearization of collagen fibers adjacent to epithelial lesions in M6-Hh tumors, a hallmark of breast tumor growth and invasiveness^8,23^ (Fig. 4a). These features were ameliorated in M6-Hh tumors treated with SMOi (Fig. 4a), demonstrating an ongoing dependency on SMO activation.

Sites of collagen deposition and crosslinking at the stromal−epithelial interface were also associated with increased epithelial phosphorylation and activation of focal adhesion kinase (FAK), a key signaling intermediate downstream of integrin receptors (Fig. 4c; Supplementary Fig. 4a). E-cadherin was used to mark the epithelial compartment (Fig. 4c, d; Supplementary Fig. 4). Increased phospho-FAK (pFAK)  and CK6 expressions were observed to occur in the same regions of the M6-Hh tumors (Fig. 4d**)**, linking fibrillar collagen content to FAK signaling and the acquisition of a stem-like phenotype in the neoplastic cells. Importantly, mechano-signaling and cancer stemness occurred primarily in dense collagen regions and were not observed in the core of M6-Hh tumors (Supplementary Fig. 4a, b).

To directly assess the sufficiency of collagen mechano-signaling to promote stemness, the clonogenic potential of M6-Ctrl and M6-Hh cells was assessed using three-dimensional cultures encapsulated within Alginate-Collagen I Inter-Penetrating Network (IPN) hydrogels. Alginates are widely used in bioengineering approaches as scaffolds^25^ and previous work has shown the successful application of IPNs for the study of fibroblast biology^26^ and the role of ECM biomechanics in the induction of malignant phenotypes in mammary epithelium^27^. In our experiments, biomechanical properties were precisely controlled through regulation of alginate gelation. The enrichment for fibrillar collagen in this in vitro model allowed the uncoupling of 3D environmental biomechanics from biochemistry, recapitulating the features of stromal collagen matrix deposition observed in Hh-expressing models. Increased content and presence of highly bundled fibrillar collagen significantly increased the clonogenic capacity of M6 cells, a functional surrogate for CSC activity, independently of Hh ligand expression (Fig. 4e). Furthermore, increasing collagen I abundance also increased expression of the stem cell markers *Id3*, *Itgb3* (CD61) and *Krt6* (CK6) **(**Fig. 4f). These data demonstrate that Hh-dependent stromal ECM remodeling is sufficient to foster a CSC phenotype.

### Paracrine Hh-FGF5 axis also contributes to CSC plasticity

**Fig 5:**
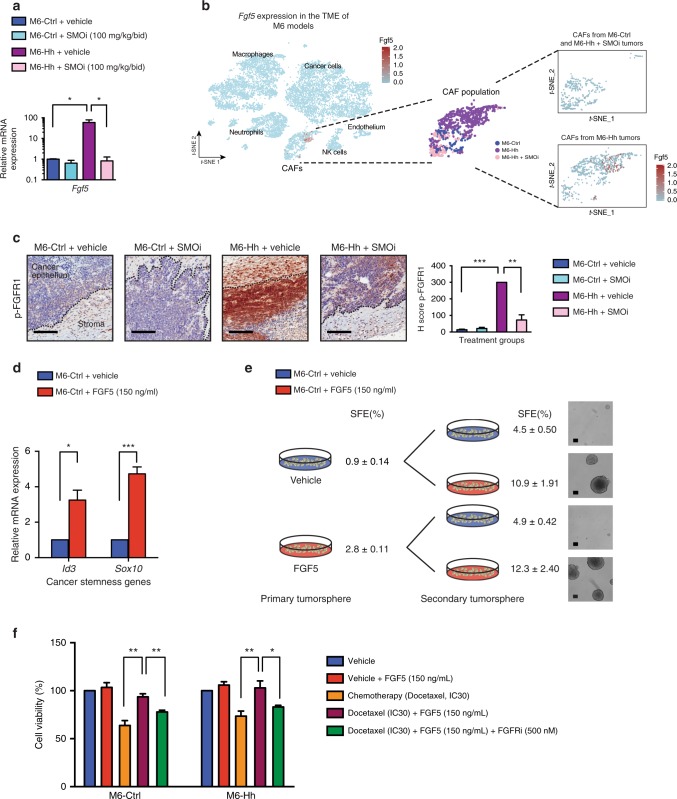
Hh-activated CAFs form a reversible, chemo-resistant CSC niche via FGF pathway activity. **a** RT-qPCR analysis of *Fgf5* expression in M6 whole tumors. *n* = 3 biological replicates per treatment group with three technical replicates per assay. Statistical significance was determined using unpaired two-tailed Student’s *t* test with equal s.d. **b** Expression of *Fgf5* at single-cell resolution in M6 tumor models. Freshly isolated M6-Ctrl (blue), M6-Hh (magenta), and M6-Hh tumors + SMOi (100 mg/kg/bid; violin dots) were captured using 10x Chromium technology. *t*-SNE plot represents the subcellular clusters present in the breast TME of M6 tumors. A sole subset of Hh-activated CAFs exhibits robust expression of *Fgf5* in M6-Hh tumors. **c** Representative immunohistochemistry staining for phospho-FGFR on M6 tumors. Scale bars: 100 μm. Quantification of phospho-FGFR-positive cancer cells at the tumor−stromal interface. *n* = 4 biological replicates per treatment group; statistical significance was determined using unpaired two-tailed Student’s *t* test with equal s.d. **d** Relative mRNA expression of CSC markers *Id3* and *Sox10* in M6-Ctrl cells treated with DMSO (vehicle; blue) or recombinant FGF5 (red) in vitro*. n* = 3 biological replicates with three technical replicates per experiment; statistical significance was determined using unpaired two-tailed Student’s *t* test. **e** Primary and secondary tumorsphere formation of M6-Ctrl cells treated with DMSO (vehicle; blue) or recombinant FGF5 (red). Sphere Formation Efficiency (SFE) values in % are mean ± s.e.m.; *n* = 3 biological replicates with three technical replicates per tumorsphere assay. Representative phase contrast micrographs of M6-Ctrl spheres upon recombinant FGF5 stimulation. Scale bars: 100 μm. **f** Cell viability of M6-Ctrl and M6-Hh cells treated with indicated agents (*n* = 5 biological replicates with six technical replicates each). Statistical significance was determined using unpaired two-tailed Student’s *t* test with equal s.d.; *P < 0.05; **P < 0.01; ***P < 0.001. Bars represent mean ± s.e.m.

To identify additional mechanisms by which stromal signaling promotes the acquisition of a CSC phenotype, we turned our attention to *Fgf5*, which was strongly upregulated in Hh-activated stroma compared to controls (Fig. 2a, c and Supplementary Data 1 and 2). qRT-PCR analysis of whole tumors confirmed ~60-fold upregulation of *Fgf5* mRNA in M6-Hh tumors, which was reversed upon SMOi treatment (Fig. 5a). Notably, a sole subset of Hh-activated CAFs exhibited robust *Fgf5* expression at single-cell resolution (*P* = 9.16×10^−12^), reflecting the close spatial localization of these CAFs with M6-Hh cells (Fig. 5b). *Fgf5* was not expressed by any of the other cell populations (Fig. 5b). Immunohistochemical analysis of phospho-FGFR revealed potent receptor activation in epithelial cells adjacent to M6-Hh tumor stroma, which was reversed upon SMOi treatment (Fig. 5c**)**. To explore the role of FGF5 in the acquisition of the CSC phenotype, M6-Ctrl cells were treated with recombinant FGF5 protein in vitro and proliferation, stem cell marker expression and sphere-forming capacity were evaluated. FGF5 treatment led to an increase in proliferation under serum and growth factor deprivation (Supplementary Fig. 5a). Importantly, the stemness markers *Id3* and *Sox10* were also robustly upregulated (Fig. 5d) and primary and secondary sphere-forming capacity increased by ~3-fold (Fig. 5e and Supplementary Fig. 5b), suggesting a specific effect of FGF5 signaling on stemness.

To test whether the induction of sphere-forming capacity by FGF5 treatment was epigenetically stable or plastic, we tested the impact of addition or removal of FGF5 to primary and secondary sphere cultures. Increased sphere formation in response to FGF5 was observed in secondary cultures regardless of whether those cells were pre-treated with FGF5 during primary cultures (Fig. 5e and Supplementary Fig. 5b). Furthermore, removal of recombinant FGF5 decreased secondary sphere formation to levels comparable to those of cells never treated with FGF5 ligand (Fig. 5e and Supplementary Fig. 5b). Consistent with this result, treatment of spheres with a small molecule inhibitor of FGFR signaling (NVP-BGJ398) prevented FGF5-mediated tumorsphere formation in primary or secondary cultures (Supplementary Fig. 5c). These results demonstrate that stromal-derived FGF5 is sufficient to promote reversible transition to a CSC phenotype, rather than through the expansion of a subpopulation of CSC.

The CSC phenotype is associated with resistance to cytotoxic chemotherapy in TNBC^28^. To test whether the FGF-dependent increase in CSC alters the sensitivity of TNBC cells to chemotherapy, the efficacy of docetaxel was evaluated in M6 cell lines in vitro. Monocultures of M6-Hh cells did not display differential sensitivity to docetaxel when compared to M6-Ctrl cells as expected (Supplementary Fig. 5d). However, stimulation of M6-Ctrl and M6-Hh models with recombinant FGF5 ligand rescued these cells from docetaxel cytotoxicity (Fig. 5f). The FGFR inhibitor NVP-BGJ 398 abrogated drug resistance conferred by FGF5 (Fig. 5f). Similar results were observed in the human MDA-MB-231 cell line model of TNBC (Supplementary Fig. 5e). This result suggests that FGF5, released by Hh-activated CAFs, may create a “chemo-resistant niche” at the tumor−stromal interface of TNBC. It also suggests that targeting both tumor and stromal compartments with chemotherapeutic regimen and SMOi, respectively, may be an effectively therapeutic strategy.

### SMOi-combined therapy improves preclinical TNBC outcomes

To directly test these findings in more clinically relevant models, we turned our analysis to xenograft models of human TNBC. All three patient-derived xenograft (PDX) models tested were Hh ligand-positive as was the MDA-MB-231 cell line model (Supplementary Fig. 6a). We found convincing evidence of exclusive stromal-restricted Hh signaling and sensitivity to SMOi, using species-specific RT-PCR (Supplementary Fig. 6b, c). In addition, in vitro treatment of MDA-MB-231 cells with SMOi did not alter Hh target gene expression or proliferation (Supplementary Fig. 6d, e), consistent with the absence of SMO expression by these cells. The HCI-002 and MDA-MB-231 models were used for further studies, as they are well-accepted models of TNBC^29^.

**Fig. 6:**
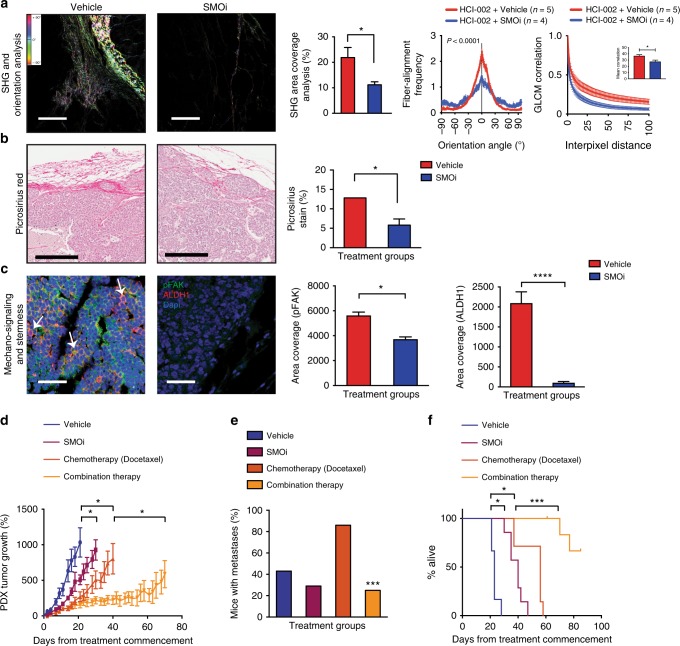
Efficacy of long-term SMOi-combined therapy in preclinical models. **a**–**c** Concomitant expression analysis of collagen content and organization, integrin pathway activation and CSC-like characteristics at the tumor−stromal interface of the TNBC HCI-002 PDX model treated with vehicle (red) or the SMO inhibitor NVP-LDE225 (80 mg/kg/day; blue) (*n* = 5 biological replicates). **a** Representative imaging (scale bars: 100 μm) and quantitative analysis of collagen abundance (left panel). Statistical significance was determined using unpaired two-tailed Student’s *t* test with equal s.d. Corresponding graphs comparing fiber orientation (middle) and quantifying GLCM analysis (right panel) in the vehicle and SMOi-treated models. Unpaired two-tailed nonparametric Mann–Whitney *U* test was used for determining statistical significance across distributions. **b** Collagen I and III deposition detected and quantified by picrosirius red staining. Scale bars, 200 μm. Statistical significance was determined using unpaired two-tailed Student’s *t* test with equal s.d. **c** Representative immunofluorescence images and quantification of concomitant phospho-FAK (green) and the human CSC marker ALDH1 (red) expression in the HCI-002 model. The white arrows illustrate examples of co-staining within the epithelial population. Scale bars, 100 μm. *n* = 3 biological replicates. Statistical significance was determined using Kruskal−Wallis test. **d**–**f** TNBC HCI-002 PDX model treated with vehicle (blue), SMO inhibitor (NVP-LDE225; 80 mg/kg/day; magenta), chemotherapy (docetaxel; 15 mg/kg/week; dark orange) or NVP-LDE225 (80 mg/kg/day) + docetaxel (15 mg/kg/week; orange line) (*n* = 7 mice per treatment group). **d** Tumor growth curves. Statistical significance was determined using unpaired Student’s *t* test. **e** Percentage of mice with detectable metastases in the lung, liver, and axillary lymph node in each treatment group. Statistical significance was determined using the Fisher’s exact test. **f** Kaplan−Meier curves of mice overall survival of each treatment group. Statistical significance was determined using Log-rank test of NVP-LDE225 + docetaxel vs. docetaxel; *P < 0.05; ***P < 0.001; ****P < 0.0001. Bars on the graphs represent mean ± s.e.m.

Analyses of the ECM in HCI-002 xenografts by SHG and picrosirius red staining revealed abundant fibrillar collagen exclusively at the tumor−stromal interface (Fig. 6a, b). The collagen fibers were highly linearized and densely packed as depicted by orientation and GLCM analyses (Fig. 6a). Areas of high fibrillar collagen density were also associated with concomitant FAK phosphorylation and expression of the human CSC marker ALDH1 in the epithelial compartment, as highlighted by co-staining experiments (Fig. 6c). As observed in the transgenic model, collagen density and orientation, FAK phosphorylation and CSC marker expression were reduced following SMO inhibition (Fig. 6a–c).

Based on these observations we predicted that SMOi would sensitize tumors to cytotoxic chemotherapy. HCI-002 PDX and MDA-MB-231 xenografts were then treated with SMOi ±  docetaxel (Fig. 6d–f). Compared with vehicle treatment, either SMOi or docetaxel monotherapy slowed tumor growth and extended mice survival (Fig. 6d, f). However, the most robust and durable therapeutic effect occurred with combined therapy (Fig. 6d, f). Interestingly, the proportion of mice with metastatic disease at ethical endpoint (based on primary tumor size) was doubled in the docetaxel treated group, an observation previously made with paclitaxel in TNBC mouse models^30^ (Fig. 6e). Combination therapy reduced the frequency of mice with metastatic disease to below that seen in the vehicle control group, despite these mice being alive much longer compared to those in the other treatment groups. Similar therapeutic benefit was observed in MDA-MB-231 tumor-bearing mice treated with combination therapy in terms of tumor growth, overall survival, and histological changes (Supplementary Fig. 7a–d).

We then examined the impact of stromal Hh pathway inhibition on the histology of the tumor epithelial and stromal compartments. The proportion and number of stromal cells in the HCI-002 PDX model were unaffected by long-term SMOi and/or chemotherapy treatments (data not shown). SMOi had no inhibitory effect on the proliferation of human cancer cells across a range of doses (Supplementary Fig. 7e). Even in the context of docetaxel chemotherapy, Hh signaling was still stromally restricted (Supplementary Fig. 7f, g), arguing that SMOi does not sensitize cells to docetaxel via a cell-autonomous mechanism.

### Clinical evaluation in the phase I clinical trial EDALINE

**Fig. 7:**
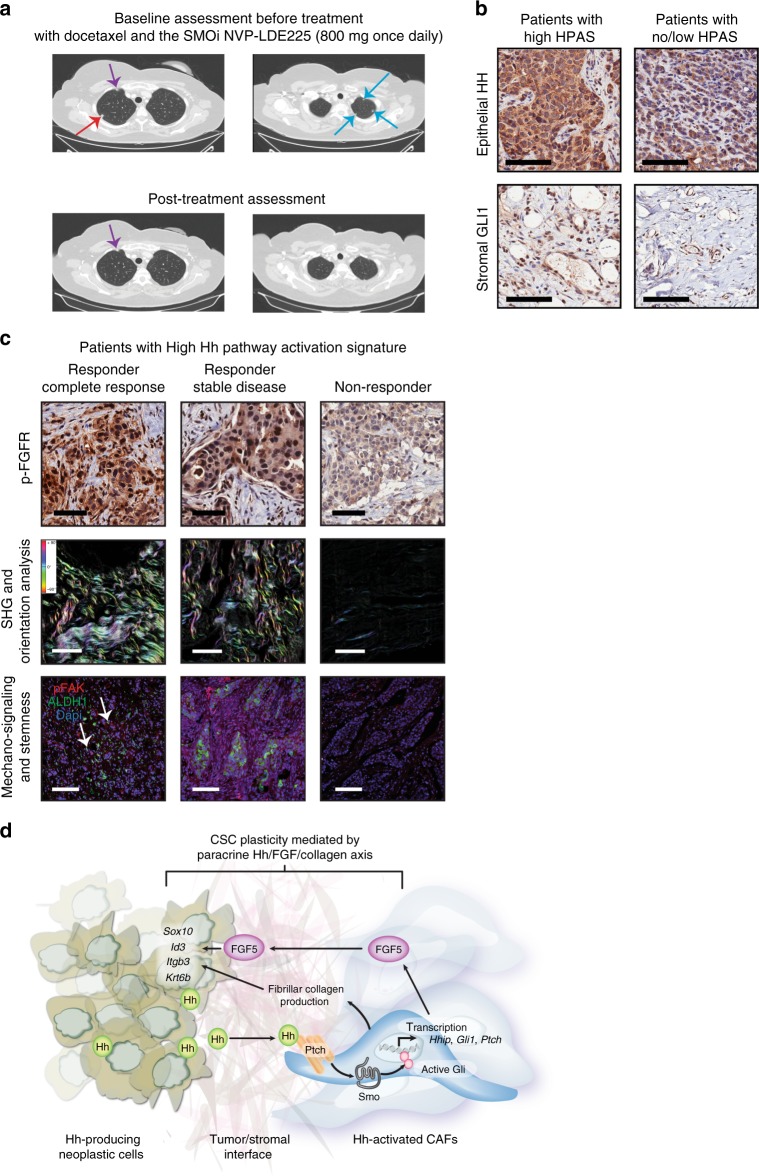
Phase I clinical trial of docetaxel and SMO inhibitor, NVP-LDE225 (EDALINE) in patients with advanced TNBC. **a** Representative computed tomography (CT) images from a patient with complete radiological response. The 54-year-old postmenopausal woman was diagnosed with recurrent metastatic TNBC in the lungs with one measurable lesion on the right upper lobe (red arrow) and several nonmeasurable lesions (blue arrows). Therapeutic response was evaluated according to RECIST criteria version 1.1. The remaining structure seen in the right upper lobe corresponds to the azygos vein (violet arrow). **b** Representative HH and GLI1 immunostaining of treatment-naive tumor specimens from patients enrolled in the EDALINE trial. The left panel is representative of patients with high HPAS (characterized by high epithelial HH ligand and high stromal GLI1 expression) while the right panel represents low/intermediate epithelial HH and low stromal GLI1 expression. Scale bars: 100 μm. **c** Representative immunohistochemistry staining for phospho-FGFR, collagen deposition depicted by SHG imaging and concomitant phospho-FAK and ALDH1 stem cell marker expression in treatment-naive tumor specimens with high HPAS from the EDALINE trial. The left panel represents tissue derived from the patient who experienced a complete clinical response, the middle is from the patient with stable disease and the right panel represents tissue from the patient with high HPAS who progressed on the prescribed regimen. Scale bars: 100 μm. The white arrows illustrate examples of co-staining. **d** Graphical summary: Paracrine Hh signaling in TNBC drives a reversible stem-like, chemo-resistant phenotype via FGF signaling and ECM remodeling. CAFs represent the primary cells of the breast TME that respond to Hh ligand stimulation. Hh-activated CAFs enhance ECM collagen deposition and express FGF5 to establish a supportive niche for chemo-resistant cancer stem cell maintenance. This study strongly highlights a novel rational approach targeting both the tumor cells and their surrounding signaling support using SMO inhibitors to overcome chemoresistance in patients with TNBC

The promising preclinical study results led us to establish the EDALINE (GEICAM/2012-12) phase I trial of docetaxel in combination with SMOi (NVP -LDE225, Sonidegib) to determine the maximum tolerated dose (MTD) and the recommended phase II dose (RP2D) of this combined therapy in patients with metastatic TNBC. Twelve patients with prior standard of care chemotherapy treatments with taxanes and/or anthracyclines were enrolled. Detailed information on clinical trial design, patient and treatment characteristics are described in Supplementary Tables 1 and 2 and on ClinicalTrials.gov (Identifier: NCT02027376). Combination therapy was well-tolerated and the RP2D of Sonidegib 800 mg once daily in combination with Docetaxel 75 mg/m^2^ every 21 days was established. Clinical response was evaluated according to standard clinical trial RECIST^31^ (Response Evaluation Criteria in Solid Tumors) criteria version 1.1. One patient experienced a complete clinical response, defined as the disappearance of all target and non-target tumor lesions and the absence of new tumor lesions. As shown in Fig. 7a, the 54-year-old postmenopausal woman presented with metastatic disease in the lungs (red and blue arrows). Following eight cycles of combined therapy, the patient achieved complete clinical response, evident by the complete resolution of all her lung metastases on routine progress CT scan (Fig. 7a and Supplementary Tables 1 and 2). Two other patients experienced disease stabilization (Supplementary Tables 1 and 2).

To assess if stromal Hh pathway activation determines clinical response, we evaluated epithelial HH ligand and stromal GLI1 expression in treatment naïve surgical tissue by immunochemistry. Only ten patient tumor samples were evaluable. Three out of ten tumors had high paracrine Hh Pathway Activation Signature (HPAS), characterized by high epithelial HH in combination with high stromal GLI1 expression (Fig. 7b and Supplementary Table 1). Of these, two patients experienced a clinical response whereas all patients with low HPAS expression had progressive metastatic disease on the prescribed treatment regimen (Supplementary Table 2). An additional patient experienced clinical benefit, but the status of Hh pathway activation was unknown as her tumor sample was not available for analysis (Supplementary Table 2).

Downstream analysis of the effect of paracrine Hh signaling revealed moderate to high phospho-FGFR expression, high collagen deposition and fiber linearization in treatment-naive tumor specimens of the two responders with biopsy material available (Fig. 7c). This correlated with elevated phospho-FAK mechano-signaling and ALDH1-positive cells at the tumor−stromal interface (Fig. 7c). In contrast, the non-responder with high paracrine HPAS exhibited weak phospho-FGFR expression, low collagen content and minimal/no evidence of mechano-signaling and breast CSCs (Fig. 7c). While the small number of patients warrants caution, these additional tumor factors deserve further investigation in future clinical trials as adjunct biomarkers of therapeutic response for patient selection for anti-SMOi-based combination therapies in Hh-expressing TNBC.

## Discussion

In certain settings, CSCs are responsible for metastasis to distant organs^32–34^ and are frequently enriched in residual tumors following chemotherapy^35–37^, reflecting a role in therapeutic resistance. The hierarchical model for CSC maintenance proposes that CSCs behave like tissue-resident physiological stem cells, self-renewing, and undergoing asymmetric divisions to generate differentiated progeny^38^. However, evidence from cell culture models has challenged the hierarchical CSC model, by suggesting that cancer cells can transition into a CSC state under specific culture conditions^38–40^. In support of this notion, we now demonstrate that stromal cues from Hh-activated CAFs, including FGF and fibrillar collagen-rich ECM, are capable of inducing and maintain a stem-like phenotype in TNBC cells in vivo. By combining a murine gain-of-function model, small molecule inhibitor studies in human xenografts with powerful in vitro systems, we have demonstrated the plastic characteristics of breast CSCs that can be successfully targeted using anti-stromal therapies, reducing metastatic growth and sensitizing to taxane chemotherapy.

Increased stromal collagen content correlates with stemness in the epidermis, both in the cancer and homeostatic contexts^41,42^. It also enhances CSC properties of breast cancer cells in vitro^43^. However, the impact of ECM collagen content and matrix mechanical properties on the biology of CSCs is not well defined. Our work provides new mechanistic insights, demonstrating that increased collagen density and fiber linearity at the tumor−stroma interface are associated with FAK activation and increased CSC number, dependent upon Hh paracrine signaling. Notably, we report a relationship between collagen abundance and clonogenicity in vitro and in vivo. Suppressing collagen production using SMO inhibitors was associated with decreased CK6^+^ and ALDH1^+^ CSCs, respectively, in both murine and human models of TNBC. Interestingly, recent data links mammographic fibrillar collagen density to breast cancer risk, raising the possibility that breast cancer progenitors in these patients may have expanded in response to a dense collagen matrix^21,44,45^. The precise mechanism controlling fibrillar collagen ECM remodeling in Hh-expressing tumors remains undetermined. Further mechanistic investigations are needed to determine if Hh induces the fibrillar collagen program in CAFs through direct regulation of collagen gene promoters via Gli1 or indirect interaction with other transcription factors, as shown in bone formation^46^.

FGF signaling has been shown to drive malignant processes including stem cell self-renewal, multipotency, and therapeutic resistance^47–50^. In metastatic breast cancer, resistance to anticancer treatment is primarily due to FGFR gene amplification^51^. Here, we demonstrate a novel ligand-driven mechanism by which FGFR activation mediates both breast cancer stemness and chemoresistance, downstream of activation of the Hh signaling pathway. Importantly, our findings suggest that CAF targeting using small molecule inhibitors of SMO is sufficient to prevent FGF ligand signaling and may overcome resistance to chemotherapies. Interestingly, FGF5 has been reported to be upregulated in prostate CAFs relative to normal fibroblasts^52^, where it is also a target of Hh-Gli signaling. Thus this axis may be operational, and of therapeutic value, in tumor types beyond TNBC.

Which effects of CAFs on cancer stemness are mediated by direct cell−cell interactions, and which by secreted factors are still unknown. Moreover, how FGFR activation and high FAK mechano-signaling lead to the establishment of a stem-like phenotype remains to be determined, but they are associated in vitro and in vivo with upregulation of transcription factors previously implicated in mammary physiological and CSCs, including ID3 and SOX10^48,53^. The mechanisms underlying *Id3* and *Sox10* transcription are unknown. *Id3* may be induced through Erk-EGR1 signaling, as observed in activated T cells, downstream of both FAK and FGFR^54^. Our data also reveal the cooperative activity of ECM remodeling and FGF signaling in driving malignancy and drug resistance, recapitulating the interaction seen between these pathways during development and wound healing^55^.

Importantly, many elements of Hh paracrine signaling to CAFs are active during embryonic development in mammals, though have not previously been linked. Dhh is highly expressed in a subset of epithelial cells of the mammary end bud, an invasive and proliferative structure responsible for ductal elongation in the developing mouse mammary gland^56^. Consistent with our observation in TNBC, stromal but not epithelial Hh signaling is required for appropriate ductal morphogenesis^57^. A number of FGF ligands are secreted by mammary stromal cells, and activation of epithelial FGFR1/2 is required for mammary ductal elongation and stem cell activity^58,59^. In addition, mammary stromal fibroblasts secrete and remodel ECM components including collagens^60^. Similar to our results in neoplastic cells, increased collagen density and mechano-signaling via FAK is sufficient to inhibit mammary epithelial cell differentiation and increased clonogenic potential^60^. Thus the paracrine Hh signaling we observe in TNBC most likely represents the dysregulation and chronic activation of a process that is important for normal mammary ductal morphogenesis.

Using high-throughput single-cell RNA-sequencing, we demonstrate that CAFs are the only stromal cell type responding to Hh ligand, and that SMO inhibitors act “on-target” to reverse CAF gene expression changes induced by Hh signaling. Surprisingly, long-term (up to 3 months) daily treatment with SMOi did not alter the stromal cell composition of mammary tumors. This result contrasts markedly to that recently observed in pancreatic, colon and bladder cancer models, where chronic SMO inhibition was associated with marked changes in stromal cellular composition and shorter survival for mice receiving long-term SMOi treatment^61–63^. The basis for this difference is not known, but may be explained by the divergent epigenomics contexts of these cancer types, resulting in the evolution of distinct TMEs^9^. Alternately, differences in the origin or phenotype of CAFs^64^ in these endodermally derived tumors vs. ectodermally derived mammary carcinomas may be relevant.

The benefit from therapeutic targeting of CAFs is twofold. Firstly, we and others have provided evidence for the crucial role of CAFs in supporting CSC self-renewal and resistance to chemotherapy^65–67^. Therefore, targeting the CAF population and the subsequent abolition of the CAF−neoplastic cell interaction represent a practical strategy to improve cancer outcomes. Secondly, unlike neoplastic cells, CAFs have not been reported to exhibit genomic instability and are therefore less likely to acquire resistance to therapy over time, making them good targets for combination cancer therapies. Combined therapy with SMOi + docetaxel was well-tolerated by mice and humans, and effective in treating a proportion of women with metastatic disease who had previously failed on taxane chemotherapy, including one patient who experienced a complete response. While patient numbers are small, these remarkable results provide the first evidence to our knowledge for clinical benefit from a CAF-directed therapy. Treatment response in patients correlated with high levels of paracrine Hh signaling, FGFR activation and fibrillar collagen deposition, suggesting that the mechanism of action in patients may be consistent with that in mouse models. Hh, FGFR or collagen pathway activation may have value as predictive biomarkers of response to SMOi. Thirty percent of TNBCs show evidence of paracrine Hh pathway activity; SMO inhibitors could then represent therapeutics of interest for a significant number of women. Whilst phase I clinical trials are not designed nor powered to assess therapeutic efficacy, these data suggest an exciting new therapeutic strategy for drug-resistant or metastatic TNBC which should proceed to prospective assessment through Phase II clinical trials.

## Methods

### Cell culture

M6 murine mammary carcinoma cells derived from the C3(1)/SV40 Tag mouse model (gift from J. Green, NIH)^68^ were cultured as previously described^6^. The human triple negative breast cancer cell line MDA-MB-231 was obtained from the American Type Cell Culture Collection (ATCC) and grown in RPMI-1640 supplemented with 10% (v/v) fetal bovine serum (FBS; Gibco^®^). All cell lines were grown at 37 °C in a 5% CO_2_ incubator. Each cell line was characterized by short tandem repeat analysis profiling using the PowerPlexR 18D System (Promega) and tested for mycoplasma contamination (MycoAlert^TM^ Mycoplasma Detection kit, Lonza). SMOi GDC-0449 (S1082, Selleckchem) and NVP-LDE225 (Novartis, Australia) were dissolved in DMSO to stock concentration of 10 mM. Docetaxel chemotherapy (McBeath, Australia) was diluted fresh (0.1 nM to 10 μM) with cell culture media. Recombinant FGF-5 (237-F5-050, R&D) was reconstituted at 10 μg/mL in PBS containing 1 μg/mL sodium heparin (H3149-50KU, Sigma-Aldrich) and 0.1% bovine serum albumin (A9418-10G, Sigma-Aldrich). NVP-BGJ 398, a potent and selective FGFR inhibitor (S2183, Selleckchem) was used at a concentration of 500 nM. All cell culture experiments involving SMOi (1 nM to 10 μM), NVP-BGJ 398 and chemotherapy (IC30) lasted 5 days and were performed in the presence of FBS. For cell viability assays, treatment of M6 cells with recombinant FGF-5 (150 ng/mL) in the absence of FBS lasted 5 days. Cell viability assays were carried out in 96-well plates (Corning^®^ Life Sciences) and were determined by alamarBlue^®^ reduction.

### Tumor dissociation

M6 tumors were processed into single-cell suspensions before limiting dilution assays, fluorescence-activated cell sorting (FACS) or flow cytometry analysis. Tumor dissociation into single-cell suspension was carried out using the MACS mouse Tissue Dissociation Kit (Miltenyi Biotec, Australia) in gentleMACS C tubes on the gentleMACS Dissociator (Miltenyi Biotec) according to the manufacturer’s recommended protocol. Briefly, up to 1.5 g of tissue was transferred into a gentleMACS C-Tube (Miltenyi Biotec) containing 2.35 mL of RPMI 1640 solution with 1× Penicillin/Streptomycin (Gibco). 100 μL of enzyme D, 50 μL of enzyme R and 12.5 μL of enzyme A were then added and the sample was processed by running the defined gentleMACS program m_impTumor_02 on the gentleMACS Dissociator. The sample was incubated for 40 min at 37 °C under continuous agitation and then processed using the gentleMACS program m_impTumour_03. The sample was resuspended in RPMI 1640 and filtered sequentially through 70 μm and 40 μm cell strainers (BD Falcon) and the resulting single-cell suspension was centrifuged at 300 × *g* for 7 min. Cells were then resuspended in 1× BD Pharm Lyse^TM^ lysing solution (555899, BD Biosciences) for 3 min at room temperature (RT) to lyse erythrocytes.

### Flow cytometry and FACS isolation

Cell sorting and flow cytometry experiments were performed at the Garvan Institute Flow Cytometry Facility. Flow cytometry was performed on a Becton Dickinson CantoII or LSRII SORP flow cytometer using BD FACSDIVA software, and the results were analyzed using Flowjo software (Tree Star Inc.). FACS experiments were performed on a FACS AriaII sorter using the BD FACSorter software. Full details of each commercial antibody used for flow cytometry and immunochemistry are described in Supplementary Table 3.

Single-cell suspensions of primary M6 tumors were incubated with anti-CD16/CD32 antibody (1:200, BD Biosciences) in FACS buffer (PBS containing salts, 2% FBS, 2% Hepes) to block nonspecific antibody binding.

For the next generation sequencing experiment, single-cell suspensions were then pelleted and resuspended in FACS buffer containing the anti-EpCAM-PerCP/Cy5.5 (1:500, BioLegend^®^, Clone: G8.8) for 20 min on ice. Cells were then washed twice in FACS buffer before being resuspended in FACS buffer containing DAPI (1:1000; Invitrogen) to discriminate dead cells. Stromal cells identified as DAPI^-^/GFP^-^/ EPCAM^−^and M6 cancer cells identified as DAPI^−^/ GFP^+^/EPCAM^+^ were collected from at least five tumor specimens per treatment group.

For the isolation of M6-Ctrl or M6-Hh cells for limiting dilution assays, cells were pelleted and resuspended in FACS buffer containing the following murine lineage markers (Lin^+^): anti-CD45-biotin (1:100; BD Pharmingen^TM^, Clone: 30-F11); anti-CD31-biotin (1:40; BD Pharmingen^TM^, Clone: 390), anti-TER119-biotin (1:80; BD Pharmingen^TM^, Clone: TER119), and anti-BP1-biotin (1:50; Affymetrix eBiosciences, Clone: 6C3) for 20 min on ice. Cells were then pelleted and resuspended in FACS buffer containing streptavidin-APC-Cy^TM^7 (1:400; BD Pharmingen^TM^) and anti-EpCAM-PerCp/Cy5.5 (1:500, BioLegend^®^, Clone: G8.8), and incubated for 20 min on ice. Cells were then washed twice in FACS buffer before being resuspended in FACS buffer containing DAPI (1:1000; Invitrogen). Live primary M6 neoplastic cells were sorted based on their GFP^+^/EpCAM^+^/Lin^−^ expression.

For the analysis of CSC properties, single-cell suspensions from M6 tumor models were incubated with the combination of the following murine lineage markers: anti-CD31-biotin (1:40), anti-CD45^-^biotin (1:100), anti-TER119-biotin (1:80), and anti-BP1^-^biotin (1:50) for 20 min on ice. Cells were then pelleted and resuspended in FACS buffer containing streptavidin-APC-Cy^TM^7 (1:400) and the following epithelial stem cell markers: anti-CD24-PE (1:500; BD Pharmingen^TM^, Clone: M1/69), anti-CD29-APC-Cy7 (1:100; BioLegend, Clone: HMβ1-1) and anti-CD61-APC (1:50; ThermoFisher), and incubated for 20 min on ice. Cells were then washed twice in FACS buffer before being resuspended in FACS buffer containing DAPI (Invitrogen).

### Coculture of primary cells

Primary M6 cells and CAFs derived from M6-Ctrl and M6-Hh tumors, respectively, were selected by FACS using the following cell surface markers: Epithelial cells: CD45^−^/CD31^−^/CD140a^−^/GP38^−^/EpCAM^+^/GFP^+^; CAFs: CD45^−^/CD31^−^/CD140a^+^/GP38^+^/EpCAM^−^/GFP^−^. After tumor dissociation, single cells were pelleted and resuspended in FACS buffer containing the following antibodies: anti-CD45-APC-eFluor^®^-780 (1:500; Affymetrix eBioscience, Clone: 30-F11), anti-CD31-biotin (1:100; BD Pharmingen^TM^, Clone: 390), anti-CD140a-APC (1:100; BioLegend^®^, Clone: APA5), anti-Podoplanin-PE (1: 1,000, BioLegend^®^, Clone: 8.1.1) for 20 min on ice. After two washes with PBS, cells were then pelleted and resuspended in FACS buffer containing Brilliant Violet 421™ Streptavidin (1:400; BioLegend®), and incubated for 20 min on ice. Cells were then washed twice in FACS buffer before being resuspended in FACS buffer containing DAPI (Invitrogen) to discriminate dead cells. Different epithelial and CAF populations from at least three tumor specimens per treatment group were isolated and cultured into 100 mm culture dishes (Corning^®^ LifeSciences) in DMEM supplemented with 10% (v/v) FBS, 50 μg/mL gentamycin and 1× antibiotic/antimycotic (15-240-096, Gibco^®^) in a 5% O_2_, 5% CO_2_ incubator at 37 °C. 10^5^ M6 cells were added to the Petri dish when CAFs derived from the corresponding tumors have reached 70% confluency and the coculture assays lasted a total of 5 days.

### Animals and surgery

All animal procedures were carried out in accordance with relevant national and international guidelines and according to the animal protocol approved by the Garvan/St Vincent’s Animal Ethics Committee (Animal ethics number 11/46). M6-Ctrl or M6-Hh (0.75×10^6^ cells/10 μL) and MDA-MB-231 (1×10^6^ cells/10 μL) transplants were carried out by surgical injection via direct visualization into the fourth mammary fat pads of pre-pubescent Rag^−^/^−^ and NOD-scid IL2rγnull (NSG) mice, respectively.

For limiting dilution studies, single-cell suspensions of viable M6-Ctrl or M6-Hh tumor cells were prepared as described in the “Tumor dissociation” section. EpCAM^+^/GFP^+^/Lin^−^ tumor cells, isolated by FACS, were transplanted in appropriate numbers into the fourth mammary fat pad of 3- to 4-week-old syngeneic Rag^−/−^ mice and aged till ethical endpoint. Extreme limiting dilution analysis (ELDA^16^) software was used to calculate the tumor-propagating cell frequency.

PDX tumor tissues, acquired from the laboratory of A. Welm^29^ were serially passaged as 2 mm^3^ fragments in the cleared fourth mammary fat pads of pre-pubescent NSG mice according to established protocols^29^. When tumors became palpable, they were measured three times weekly in a blinded manner using electronic calipers to monitor growth kinetics. Tumor volume was calculated using the formula (π/6) × length × width^2^. Upon reaching ethical or predefined experimental endpoints, mice were euthanized and primary tumor and any associated metastases were collected.

### In vivo drug treatment experiments

SMOi GDC-0449 (S1082, Selleckchem) and NVP-LDE225 (Novartis, Australia) were dissolved in 0.5% methylcellulose, 0.2% Tween^®^ 80 (Sigma-Aldrich) and 0.5% methylcellulose, 0.5% Tween^®^ 80, respectively, and then delivered by oral gavage (100 mg/kg/bid, GDC-0449; 80 mg/kg/day, NVP-LDE225). Chemotherapy (Docetaxel, McBeath Australia) was diluted in 5% dextrose then delivered by intraperitoneal injection (15 mg/kg/week). Tumor-bearing mice were randomly assigned into respective treatment groups once tumor volume reached 100 mm^3^ (*n* = 7–8 mice per group). Tumor growth was calculated for each individual tumor by normalizing to the tumor volume at day 0. In short-term studies examining the molecular and histological impact of Hh pathway activation and inhibition, mice were treated between 8 and 14 days then euthanized. At euthanasia, primary tumors were harvested and macroscopic metastatic lesions were scored. For the long-term therapeutic study, mice were treated to endpoint. Animals were excluded from overall survival analysis if they had to be sacrificed for poor body conditioning, unrelated to tumor size endpoint. Animal technicians, who were blinded to the experiment treatment groups, independently monitored the mice.

### Next generation sequencing

We isolated by FACS the stromal DAPI^−^/GFP^−^/EPCAM^−^and epithelial DAPI^−^/ GFP^+^/EPCAM^+^ cell fractions from at least five M6-Ctrl and M6-Hh tumor models treated with vehicle (0.5% methylcellulose, 0.2% Tween^®^ 80) or with SMOi. RNA was isolated using the miRNeasy kit (Qiagen). For standard input samples, 1 μg of total RNA was used as input to the TruSeq RNA Sample Preparation Kit v2 (Illumina). The samples were prepared according to the manufacturer’s instructions, starting with the poly-A pulldown. The number of PCR cycles was reduced from 15 to 13, to minimize duplications. The samples were sequenced on the HiSeq2000 using v3 SBS reagents (Ramaciotti Centre for Genomics, University of New South Wales (UNSW)). Low-input RNA stromal samples were firstly amplified using the Ovation^®^ RNA-Seq System V2 kit (4 ng of total RNA input; Nugen Integrated Sciences Pty. Ltd.) according to the manufacturer’s instructions. 1 μg of the cDNA was sheared with Covaris to fragment sizes of ~200 bp. The material was used as input to the TruSeq RNA Sample Preparation v2 kit, starting at the end-repair step. The number of PCR cycles was reduced from 15 to 10. All the samples were sequenced on the HiSeq2000 using v3 SBS reagents (Ramaciotti Centre for Genomics, University of New South Wales).

### Bioinformatics and computational analysis

Analysis of the RNA-Sequencing data was conducted on the high-performance computing cluster at the Garvan Institute following a standard four-step approach, cleaning, aligning, counting, and differential expression with an additional normalization step. FASTQ files were quality checked using FastQC version 0.11.1 (http://www.bioinformatics.babraham.ac.uk/projects/fastqc/) and FastQ Screen version 0.4.4 (http://www.bioinformatics.babraham.ac.uk/projects/fastq_screen/) then quality filtered using FastqMCF version 1.1.2 (https://code.google.com/p/ea-utils/wiki/FastqMcf) to remove low quality bases and adapter contamination. Filtered reads were then aligned to the mouse reference genome GRCm38/mm10 using STAR, version 2.4.0d . Feature counting was performed using HTSeq version 0.5.4p3 . Due to high levels of variation in the expression data between replicates, the RUV normalization procedure was implemented^69^. This aims to remove unwanted variation and produce more reliable pair-wise comparisons when calculating differential expression. In this instance, RUVr with a K of 3 was found to be the most effective method based on the suggested diagnostics, e.g. plots of *P* value distributions and PCA. Differential expression analysis was performed within the RUV analysis using edgeR^70^.

### Single-cell RNA-Sequencing using the Chromium Platform

M6 tumors were processed into single-cell suspensions as described previously. Sorted live single epithelial and stromal cells were loaded on a Chromium Single Cell Instrument (10× Genomics, Pleasanton, CA) to generate single-cell Gel Bead-in-Emulsion (GEMs). As per the manufacturer’s instructions, approximately ~7000 cells were loaded per channel for a target of ~4000 cells (10× Genomics). Two biological replicates were analyzed per sample. Single-cell RNA-Seq libraries were prepared using the Chromium Single Cell 30 Gel Bead and Library Kit (10× Genomics). Single-cell sequencing libraries were generated from sorted cells collected in parallel on the same Chromium Single Cell Chip and sequenced in multiplexed pairs to minimize experimental variability and any confounding batch effects. Single-cell libraries were sequenced using the Illumina NextSeq 500 system using the following parameters: pair-end sequencing with dual indexing, 26 cycles for Read1, 8 cycles for I7 Index Read and 98 cycles for Read2.

The Cell Ranger Single Cell Software v2.0 (10× Genomics) was used to process raw bcl files to perform sample demultiplexing, barcode processing and single-cell 3′ gene counting (https://software.10xgenomics.com/single-cell/overview/welcome). Reads were mapped to the mm10 mouse reference genome. The Cell Ranger aggregation pipeline was used to normalize the sequencing depths of multiple datasets (based on the proportion of mapped reads) to recompute a combined gene−cell barcode matrix.

Downstream filtering and analysis of the raw gene−cell barcode matrix was performed using the Seurat v2.0 package in R^71^. The gene−cell barcode matrix was filtered based on number of genes detected per cell (any cells with less than 500 or more than 6000 genes per cell were filtered), a total number of unique molecular identifiers (UMIs) (any cells with UMIs > 50,000 were filtered) and a percentage of mitochondrial UMI counts (any cells with more than 10% of mitochondrial UMI counts were filtered). Altogether, in the combined five datasets, a total of 14,950 cells and 18,487 genes were analyzed. Based on an expression cut-off of 0.0125 and a dispersion cut-off of 0.5, a total of 2620 variable genes were selected for principal component analysis (PCA). A total of 91 significant principal components (determined using JackStraw in Seurat, *P* < 0.05) were used for clustering analysis and *t*-SNE projection. The classification of cell clusters was inferred using the following canonical markers: CAFs (*Pdpn* and *Pdgfrb)*, epithelial cancer cells (*Epcam)*, endothelial cells (*Cd34* and *Pecam1*), macrophages/monocytes (*Ptprc* and *Cd68*), neutrophils (*Ptprc* and *Csf3r*), and natural killer cells (*Ptprc* and *Ncr1)*. Differential gene expression analysis in Seurat was performed using the “bimod” likelihood-ratio test.

### Gene set enrichment analysis

Gene-sets used in GSEA were extracted from version 3.1 and 4.0 of the Broad Institute’s Molecular Signatures Database (MSigDB) and extended with additional curated gene-sets from literature. All GSEA analyses were performed using a combined set of the c2, c5 and c6 from MSigDB plus additional curated sets that we identified in the literature.

### Association of gene signatures with clinical outcome

A stringent mouse gene signature was derived through differential expression analysis of the stromal fraction of M6-Hh tumors in comparison to the stroma of M6-Ctrl or M6-Hh tumors treated with SMOi. The stromal mouse gene signature was then converted to human genes using NCBI homolog gene list v68 (http://www.ncbi.nlm.nih.gov/homologene). The converted gene list consists of 146 upregulated genes and 39 downregulated genes in the Hh-activated stromal population (Supplementary Data 1). The gene list was further assessed for survival analysis using the TCGA breast invasive carcinoma cohort. The processed TCGA data were downloaded from cBioPortal^72^ based on the TCGA study^19^. The gene signature score was defined by a weighted average method for each sample in the TCGA cohort. Survival curves were estimated using the Kaplan−Meier method, with overall survival used as the outcome metric.

### RNA isolation and quantitative RT-PCR experiments

Individual stromal CAFs and epithelial malignant M6 cancer cells were FACS-isolated as described above. For each fraction, between 1,000 and 50,000 cells were directly collected into QIAzol lysis reagent. Low-input RNA samples were then isolated using the miRNeasy Micro kit (Qiagen). All the other standard input RNA samples were isolated using the miRNeasy kit (Qiagen) and reverse transcribed with the Transcriptor First Strand cDNA Synthesis Kit (Roche Diagnostics). cDNA was synthesized from 0.5 to 2 μg of total RNA and diluted 1:10 before any further quantitative RT-PCR (qRT-PCR) analysis.

qRT-PCR experiments were performed using either the Roche Universal Probe Library System on a Roche LightCycler480^®^ (Roche LifeScience) or the TaqMan Gene Expression Assay (Applied Biosystems/Life Technologies) on an ABI Prism^®^ 7900 HT Sequence Detection System (Perkin-Elmer Applied Biosystems). Primers, probes, and programs used for qRT-PCR analysis are listed in Supplementary Table 4. Relative mRNA expression levels were normalized to β-actin, GAPDH, or HPRT and quantification was performed using the comparative *C*_T_ method described by Livak and Schmittgen^73^.

### Histological assays and analysis

Tissues were fixed in 10% neutral buffered formalin at 4 °C overnight then processed for paraffin embedding. For histological analysis, 4 μm tissue sections were stained with hematoxylin and eosin using standard methods. Immunohistochemical, immunofluorescence, and picrosirius red staining were performed on paraffin-embedded tissue sections using standard protocols. Full details of each antibody used and their relative staining protocols for immunochemistry are described in Supplementary Table 3.

Histological analysis of the proliferative marker phospho-histone H3, ALDH-1, and the progenitor cell marker CK6 were carried out by digitizing entire images using the Aperio CS2 digital pathology slide scanner (Leica Biosystem) at ×20 magnification. Cells that stained positively for phospho-Histone H3, ALDH-1, or CK6 within a distance of 200 μm from CAFs at the tumor−stromal interface were then counted and averaged over at least five fields using the Aperio Imagescope software (Leica Biosystem). The limit of 200 μm reflects the well-established diffusional distance for Hh ligand in mammalian models^74^. Picrosirius red stain was analyzed as previously described^75^. Two specialist breast pathologists, who were blinded to the experiment treatment groups, independently scored the remaining IHC stains. Areas of necrosis were excluded for all analyses. The number of CD45^+^ cells in the peritumoral stroma and the number of CD31^+^ blood vessels were estimated and averaged over five high power fields (×40 magnification). Alpha-SMA was scored as the percentage of myofibroblabts in the tumor stroma at the whole tumor level. Tumor vs. stromal ratio was estimated on H&E sections.

For immunofluorescence staining, formalin-fixed paraffin-embedded (FFPE) sections on Superfrost+ glass slides (Thermo) were dewaxed and rehydrated, and antigen retrieval was performed by boiling for 12 min in 10 mM citrate buffer (pH 6.0) or 10 mM Tris-Cl (pH 9.0) in a pressure cooker (Supplementary Table 3). Following blocking with 10% goat serum, sections were incubated with the primary antibodies p(Tyr397)-FAK, CK6, ALDH1, E-cadherin at 4 °C for 18 h, washed in PBS and incubated with the appropriate secondary antibodies for 1 h. After washing in PBS, sections were incubated with 0.2 µg/mL DAPI in PBS for 5 min to label nuclei and mounted. Confocal fluorescence images were captured using an LSM 700 confocal scanning system (Carl Zeiss AV), with Zen 2011 (Black Edition) version 8.1.5.484 software. Images were processed using ImageJ (National Institutes of Health, Bethesda MD, USA) as previously described^42^. Two cell biologists from separate institutes, who were blinded to the treatment groups, independently scored the IF stains for CK6, ALDH1, and phospho-FAK.

### Microscopy analyses of collagen deposition and organization

Formalin-fixed, paraffin-embedded sections stained with hematoxylin and eosin and mounted in DPX (Sigma) were imaged using a ×20 1.0 NA objective on an upright fixed-stage two-photon laser scanning microscope system (Zeiss). The excitation source was a Ti:Sapphire femto-second laser cavity (Newport Mai Tai), coupled into an LSM 710 scan module. An excitation wavelength of 890 nm was used to collect SHG signal (435 ± 20 nm) from collagen. Maximum collagen coverage values derived from SHG signal (by depth (line graph) and at peak value (histogram inset)) was used as a measure of collagen abundance and density. Signal was acquired from three separate areas measuring 320 × 320 μm^2^ across each sample. Bright-field transmission images were co-acquired with SHG data.

ImageJ was used to calculate percentage area covered by SHG signal per image, after conversion to a binary image based upon a single manually determined threshold value applied across all images as previously described^41^. Results were expressed as medians, ranges, and quartiles across all datasets.

Stromal collagen fiber organization and crosslinking were assessed using GLCM^24^ analysis to characterize the texture of a sample and determine the correlation of the SHG signal within the matrix. The correlation plots represent the similarity in signal strength between pixels. A slower decay shows a more organized and correlated network of collagen fibers than in samples with a faster decay. GLCM analysis was performed in ImageJ.

Orientation analysis was carried out as previously described^23^. Briefly, fiber orientation analysis was performed on SHG images using an in-house ImageJ macro where structure tensors were derived from the local orientation and isotropic properties of pixels that make up collagen fibrils. Within each input image, these tensors were evaluated for each pixel by computing the continuous spatial derivatives in the *x* and *y* dimensions using a cubic B-spline interpolation. From this, the local predominant orientation was obtained. The peak alignment (measured in degrees) of fibers was then determined, and the frequency of fiber alignment calculated.

### Alginate-Collagen I Inter-Penetrating Network hydrogels

Alginate-Collagen I Inter-Penetrating Network (IPNs) hydrogels were generated from 1% sodium alginate mixed with either 20, 5 or 0% rat tail collagen I, plus 1% Matrigel. A standard guide describing cell encapsulation in alginate is available from ASTM International (ASTM F2315–11). Well-characterized alginates with high purity and G-block composition were used to prepare hydrogels with consistent mechanical properties for cell encapsulation. M6-Ctrl and M6-Hh single cells were encapsulated in 400 μm diameter beads before transferring to normal growth media. Colony-forming ability was assessed at days 5, 8, and 12.

### Tumorsphere assays

Low passage M6 cells grown to 70–80% confluency as adherent monolayer were trypsinized, quenched in normal culture media, washed three times with large volumes of calcium-magnesium-free PBS then passed through a 40 μm cell strainer to obtain a single-cell suspension. Cell number was determined using the Countess™ Automated Cell Counter (Invitrogen) then seeded in sphere-promoting culture at a density of 2.5×10^3^ cells/mL in ultra low-adherent six-well plates (Corning^®^ LifeSciences). Cells were grown at 37 °C in a 5% CO_2_ incubator. Primary sphere formation efficiency was determined after 5 days. Spheres larger than 40 μm were counted manually using a light microscope and automatically using the IncuCyte ZOOM^®^ Live Cell System (Essen BioScience). Primary spheres were then collected by gentle centrifugation and washed with calcium-magnesium-free PBS prior to dissociation into single-cell suspension. Cell number was determined as above then seeded in triplicate at a density of 5×10^2^ cells/mL in ultra low-adherent six-well plates. Secondary sphere formation efficiency was determined after 8 days. Sphere media was composed of DMEM/F12, 1% methylcellulose, 1× B27 supplement (17504-044, Invitrogen), and 4 μg/mL sodium heparin (H3149-50KU, Sigma-Aldrich). Tumorspheres were treated with 150 ng/mL recombinant FGF5 (237-F5-050, R&D Systems) and/or 500 nM FGFR inhibitor, NVP-BGJ 398 (S2183, Selleckchem). Sphere formation efficiency was calculated using the following formula: (Number of tumorspheres larger than 40 μm / Number of single cells seeded) × 100%.

### Patients

Patients in this study were enrolled in the GEICAM/2012-12 (EDALINE) clinical trial (ClinicalTrials.gov Identifier: NCT02027376); a single-arm, open-label, phase I, 3 + 3 dose escalation study in which patients with TNBC were treated with the SMOi Sonidegib (LDE-225) in combination with docetaxel to determine the MTD and the Recommended Phase II Dose (RP2D), as the primary objective. Written informed consent was obtained from all patients and documented before performing any protocol-specific procedure. The study was conducted in accordance with the International Conference on Harmonization Good Clinical Practice Guidelines (ICH GCP), the Declaration of Helsinki and applicable local regulatory requirements and laws. The protocol was approved by the Institutional Review Board and the Ethics Committee of all the participating sites (Hospital General Universitario Gregorio Marañón, Hospital Universitario Clínico San Carlos, Complejo Hospitalario Universitario A Coruña, Hospital Universitario Virgen del Rocío, Hospital Universitario Virgen de la Victoria), according to the requirements of the Spanish regulations (GEICAM/2012-12; clinicaltrials.gov identifier: NCT02027376). Eligible patients, with no more than three previous lines of chemotherapy for metastatic disease were treated with 21-day cycles of intravenous docetaxel 75 mg/m^2^ on day 1 and oral Sonidegib administered at increasing doses of 400, 600, and 800 mg once daily (QD), until radiographic or symptomatic progression, unacceptable toxicity or withdrawal of the informed consent, whichever occurred first. All patients at each dose level completed at least two treatment cycles before being enrolled to the next Sonidegib dose level. Twelve patients were enrolled into the study and were treated as described above.

### Clinical markers of therapeutic activity

The expression of Hedgehog ligand (HH) and GLI1 were examined by immunohistochemistry (IHC) from archived FFPE pretreatment primary breast tumors from patients enrolled in the EDALINE clinical trial. Pathologists, who were blinded to clinical parameters, carried out biomarker analysis. Two specialist breast pathologists independently calculated the Histo-score (Hscore) based on the percentage of stained cells (HH and GLI1 expression) and staining intensity on a predetermined scale (0: no staining to 3: strong), in the tumor epithelial cells (HH) and the tumor stroma (GLI1). Predefined cut-offs for high/low biomarker expression were established based on standard criteria (median Hscore for HH in tumor cells and intensity staining for GLI1 in the stroma).

We defined an HPAS predictive for clinical response to sonidegib (LDE-225) in combination with docetaxel as cases with both high HH expression in the tumor epithelium (HH Hscore > 150) and intense GLI1 expression in the tumor stroma.

### Statistical analysis

All statistical analyses were performed using GraphPad Prism 6.0c software (GraphPad Software). For all in vitro experiments, three or six technical replicates were analyzed for each experiment, and results are presented as the mean ± s.e.m. of three biological replicates. Quantitative analyses were carried out using unpaired two-tailed Student’s *t* test with equal standard deviation after confirming that the data met appropriate assumptions (normality, homogenous variance, and independent sampling). Seven to ten mice per treatment group were used for all in vivo experiments with SMOi treatment. Five mice per treatment group were utilized for RNA-Seq. For all RT-qPCR experiments, three technical replicates were analyzed for each experiment, and results are presented as the mean ± s.e.m. of three biological replicates. Subsequent statistical analyses of in vivo experiments were performed with either unpaired two-sided Student’s *t* tests or the Fisher’s exact test. Survival analysis was performed using the Log-rank (Mantel−Cox) test. A *P* value < 0.05 was considered statistically significant. *P < 0.05, **P < 0.01, ***P < 0.001, ****P < 0.0001. Estimation of variation within each group was determined by s.e.m. Sample size estimation was initially chosen by using power calculations for guidance (http://biomath.info/power/ttest.htm). Effect sizes were estimated at 30% for single agent arms, based on earlier data with single agent docetaxel and SMO inhibitors. With alpha = 0.05 and power = 0.9 and allowing for attrition of ~1 mouse/group, ≥7 mice per group were needed for all preclinical studies. For the Phase I clinical trial EDALINE, descriptive analysis on demographic and clinicopathological characteristics (age, visceral disease, number of involved sites, prior treatment, histologic tumor grade and Ki67), Hh biomarkers expression (HH and GLI1) and efficacy data to the combined therapy sonidegib with docetaxel were performed. The Chi-square test of Independence was assessed to examine the association between epithelial HH and stromal GLI1 expression and efficacy endpoints: best tumor response (complete or partial response, stable disease, progression disease, and Time to Progression (TTP)). TTP was defined as the time from treatment commencement until objective tumor progression (does not include deaths). Progression-free survival (PFS) was explored using Kaplan−Meier survival analysis. PFS was defined as the time from treatment commencement until disease progression or death. For mouse and clinical studies, specialists were blinded to the experiment treatment groups.

### Data availability

All relevant data are available from the authors. All RNA-Sequencing files that support the findings of this study have been deposited in GEO with the accession code PRJNA369574. The RNA-Seq pipeline and the analysis scripts can be found on the respective websites: https://github.com/elswob/rna-seq-pipe and https://github.com/elswob/Hh.

## Electronic supplementary material

Supplementary Information

Description of Additional Supplementary Files

Supplementary Data 1

Supplementary Data 2
