# Supplementary material for: Targeting stromal remodeling and cancer stem cell plasticity overcomes chemoresistance in triple negative breast cancer
Source: Nat Commun. 2018 Jul 24;9:2897. doi: 10.1038/s41467-018-05220-6 (PMC6057940; doi:10.1038/s41467-018-05220-6)
Supplement: Supplementary file 1 — Supplementary Information [file 41467_2018_5220_MOESM1_ESM.pdf]

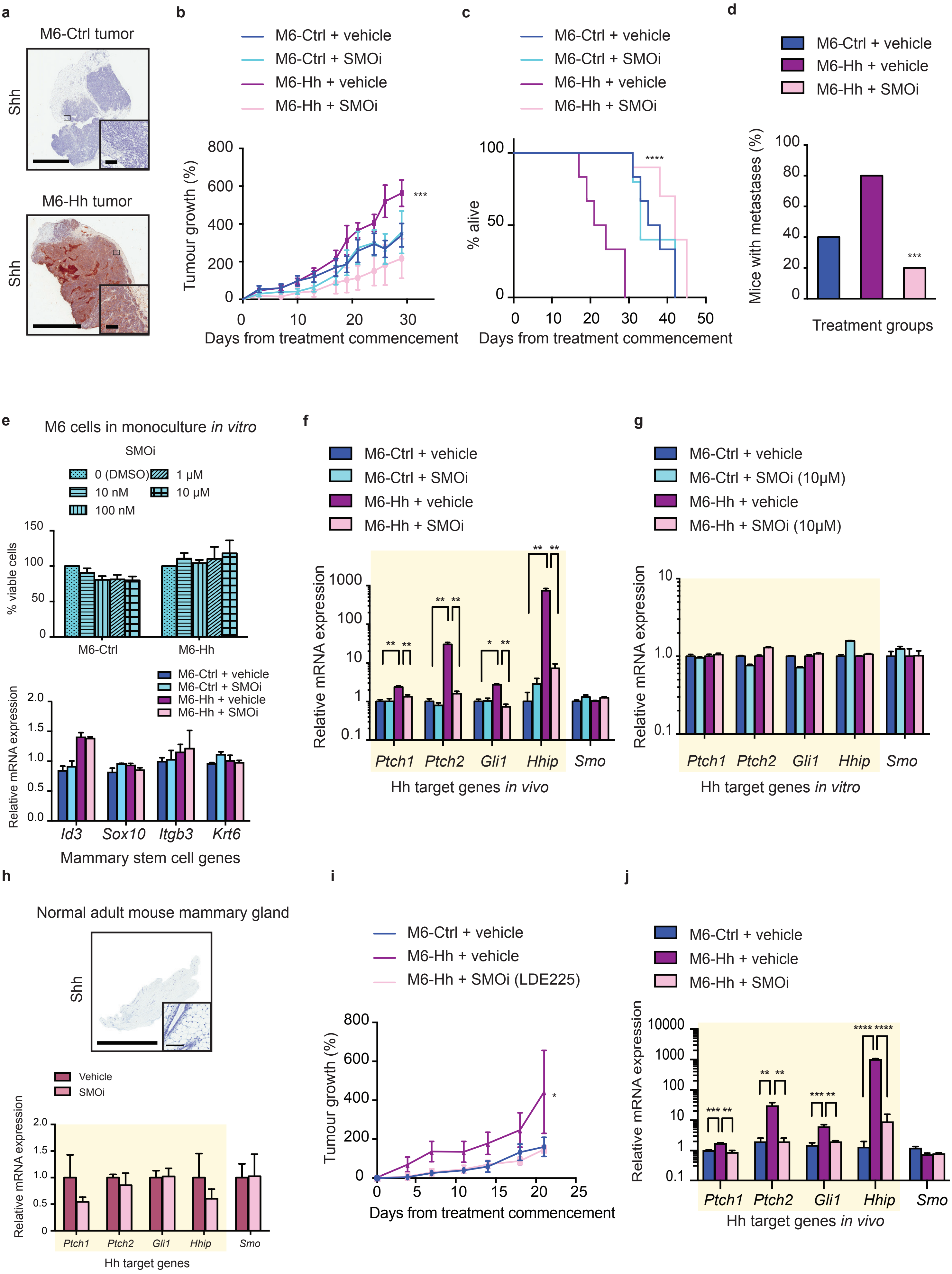
a
M6-Ctrl tumor
Shh
M6-Hh tumor
Shh
b
M6-Ctrl + vehicle
M6-Ctrl + SMOi
M6-Hh + vehicle
M6-Hh + SMOi
Tumour growth (%)
800
600
400
200
0
***
0
10
20
30
Days from treatment commencement
c
M6-Ctrl + vehicle
M6-Ctrl + SMOi
M6-Hh + vehicle
M6-Hh + SMOi
% alive
100
50
0
****
0
10
20
30
40
50
Days from treatment commencement
d
M6-Ctrl + vehicle
M6-Hh + vehicle
M6-Hh + SMOi
Mice with metastases (%)
100
80
60
40
20
0
***
Treatment groups
e
M6 cells in monoculture in vitro
SMOi
0 (DMSO)
1 μM
10 nM
10 μM
100 nM
% viable cells
150
100
50
0
M6-Ctrl
M6-Hh
M6-Ctrl + vehicle
M6-Ctrl + SMOi
M6-Hh + vehicle
M6-Hh + SMOi
Relative mRNA expression
2.0
1.5
1.0
0.5
0.0
Id3
Sox10
Itgb3
Krt6
Mammary stem cell genes
f
M6-Ctrl + vehicle
M6-Ctrl + SMOi
M6-Hh + vehicle
M6-Hh + SMOi
Relative mRNA expression
1000
100
10
1
0.1
** **
** **
* **
** **
Ptch1
Ptch2
Gli1
Hhip
Smo
Hh target genes in vivo
g
M6-Ctrl + vehicle
M6-Ctrl + SMOi (10μM)
M6-Hh + vehicle
M6-Hh + SMOi (10μM)
Relative mRNA expression
10
1.0
0.1
Ptch1
Ptch2
Gli1
Hhip
Smo
Hh target genes in vitro
h
Normal adult mouse mammary gland
Shh
Vehicle
SMOi
Relative mRNA expression
2.0
1.5
1.0
0.5
0
Ptch1
Ptch2
Gli1
Hhip
Smo
Hh target genes
i
M6-Ctrl + vehicle
M6-Hh + vehicle
M6-Hh + SMOi (LDE225)
Tumour growth (%)
800
600
400
200
0
*
0
5
10
15
20
25
Days from treatment commencement
j
M6-Ctrl + vehicle
M6-Hh + vehicle
M6-Hh + SMOi
Relative mRNA expression
10000
1000
100
10
1
0.1
*** **
** **
*** **
**** ****
Ptch1
Ptch2
Gli1
Hhip
Smo
Hh target genes in vivo

Supplementary Figure 1. Discovery model: Impact of SMOi on a mouse model of basal-like mammary carcinoma

(a) Representative Shh immunostaining of M6-Ctrl and M6-Hh tumors. Insets show magnified view of sections marked by the box. Scale bars: 2 mm (main panels), 100 µm (insets). **(b)** Growth of M6-Ctrl and M6-Hh tumors treated with vehicle or the SMOi, GDC-0449 (Vismodegib, SMOi; 100 mg/kg/bid; *n* = 6 mice per treatment group). Statistical significance was determined between M6-Hh tumors treated with vehicle or SMOi using unpaired two-tailed Student's t-test with equal s.d; *** $P < 0.001$. **(c)** Kaplan-Meier curves of mice overall survival of each treatment group. *n* = 6 biological replicates per treatment group. Statistical significance was determined using the Log-rank test of M6-Hh tumor bearing mice treated with SMOi (100 mg/kg/bid) versus vehicle; **** $P < 0.0001$. **(d)** Percentage of mice with detectable metastases in the lung, liver, pancreas and axillary lymph node in each treatment group. *n* = 6 mice per treatment group. Statistical significance was determined using Mann-Whitney test; *** $P < 0.001$. **(e)** Top panel: cell viability of M6-Ctrl and M6-Hh cells treated with increasing doses of SMOi *in vitro* (*n* = 3 biological replicates with 6 technical replicates per treatment group). Bottom panel: real time PCR measurement of mammary CSC markers in primary M6-Ctrl and M6-Hh cells in mono-culture *in vitro* (*n* = 4 biological replicates per group). **(f)** Real time PCR measurement of Hh target genes in M6-Ctrl and M6-Hh tumors treated with vehicle or SMOi (100 mg/kg/bid; *n* = 3 biological replicates per treatment group). Statistical significance was determined using unpaired two-tailed Student's t-test with equal s.d; * $P < 0.05$; ** $P < 0.01$. **(g)** Real time PCR measurement of Hh target genes in M6-Ctrl and M6-Hh cells treated with vehicle or SMOi *in vitro* (10 µM; *n* = 3 biological replicates with 3 technical replicates each). **(h)** The Hh signaling pathway is quiescent in benign murine mammary gland. Representative Shh immunostaining of benign murine mammary gland tissue from $Rag^{-/-}$ mice (top panel). Scale bars: 2 mm (main panel), 100 µm (inset). Real time PCR measurement of Hh target genes in benign murine mammary glands treated with SMOi (100 mg/kg/bid; bottom panel; *n* = 5 biological replicates per treatment group). **(i)** Growth of M6-Ctrl and M6-Hh tumors ± the SMOi LDE-225 (Sonidegib, 80 mg/kg/day; *n* = 6 mice per treatment group). Statistical significance was determined between M6-Hh tumors treated with vehicle or LDE-225 using unpaired two-tailed Student's t-test with equal s.d; * $P < 0.05$. **(j)** Real time PCR measurement of Hh target genes in M6-Ctrl and M6-Hh tumors treated with vehicle or LDE-225 (*n* = 3 biological replicates per treatment group). Statistical significance was determined using unpaired two-tailed Student's t-test with equal s.d; * $P < 0.05$; ** $P < 0.01$; *** $P < 0.001$ and **** $P < 0.0001$. Bars represent mean ± s.e.m.

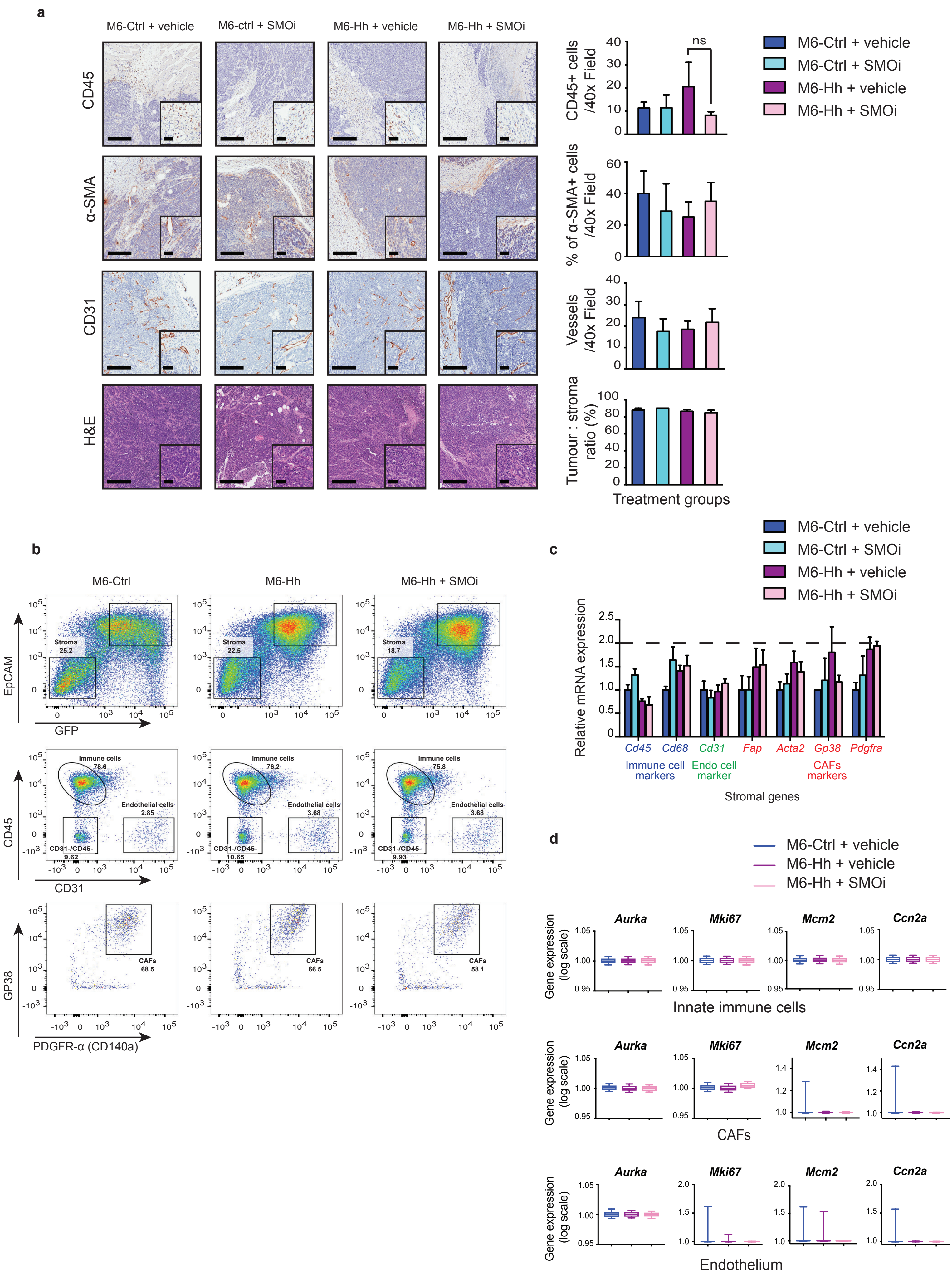

a
M6-Ctrl + vehicle
M6-ctrl + SMOi
M6-Hh + vehicle
M6-Hh + SMOi
CD45
α-SMA
CD31
H&E
CD45+ cells /40x Field
ns
% of α-SMA+ cells /40x Field
Vessels /40x Field
Tumour : stroma ratio (%)
Treatment groups
M6-Ctrl + vehicle
M6-Ctrl + SMOi
M6-Hh + vehicle
M6-Hh + SMOi
b
M6-Ctrl
M6-Hh
M6-Hh + SMOi
EpCAM
GFP
Stroma 25.2
Stroma 22.5
Stroma 18.7
CD45
CD31
Immune cells 78.6
Immune cells 76.2
Immune cells 75.8
Endothelial cells 2.85
Endothelial cells 3.68
Endothelial cells 3.68
CD31-/CD45- 9.62
CD31-/CD45- 10.65
CD31-/CD45- 9.93
GP38
PDGFR-α (CD140a)
CAFs 68.5
CAFs 66.5
CAFs 58.1
c
Relative mRNA expression
Cd45
Cd68
Cd31
Fap
Acta2
Gp38
Pdgfra
Immune cell markers
Endo cell marker
CAFs markers
Stromal genes
d
M6-Ctrl + vehicle
M6-Hh + vehicle
M6-Hh + SMOi
Gene expression (log scale)
Aurka
Mki67
Mcm2
Ccn2a
Innate immune cells
CAFs
Endothelium

Supplementary Figure 2. SMO-targeted treatment does not alter stromal cell composition in M6 primary tumors

(a) Representative images of immunostaining for immune cells (CD45), CAFs (α-SMA), endothelial cells (CD31) and representative hematoxylin and eosin (H&E) images of the tumor:stroma ratio. Scale bars: 200 μm (main panels), 100 μm (insets). Quantitative analysis of immune cells, CAFs, endothelial cells and the tumor:stroma ratio (*n* = 4 biological replicates per treatment group). Bars represent mean ± s.e.m. **(b)** Relative abundance of different stromal cell types (GFP^{-}/$EpCAM^{-}$) from freshly-isolated M6 tumors analyzed by immunostaining and flow cytometry: immune cells ($CD45^{+}$), endothelial cells ($CD31^{+}$) and CAFs ($GP38^{+}$/ PDGFR-α^{+}). Representative histograms from three independent experiments. **(c)** Real time PCR measurement of specific transcript markers of stromal lineages from whole tumors: immune cells (*Cd45* and *Cd68*), endothelial cells (*Cd31*) and CAFs (*Fap*, *Acta2*, *Gp38*, *Pdgfra*) (*n* = 3 mice per treatment group with 3 technical replicates per treatment group). Bars represent mean ± s.e.m. **(d)** Expression of markers of cell proliferation from single cell analysis of different stromal components. A total of 6,649 innate immune cells, 276 endothelial cells and 1,182 CAFs were analyzed within the breast TME of M6-Ctrl, M6-Hh and M6-Hh + SMOi tumors. Normalized RNA expression values ± s.e.m. are shown.

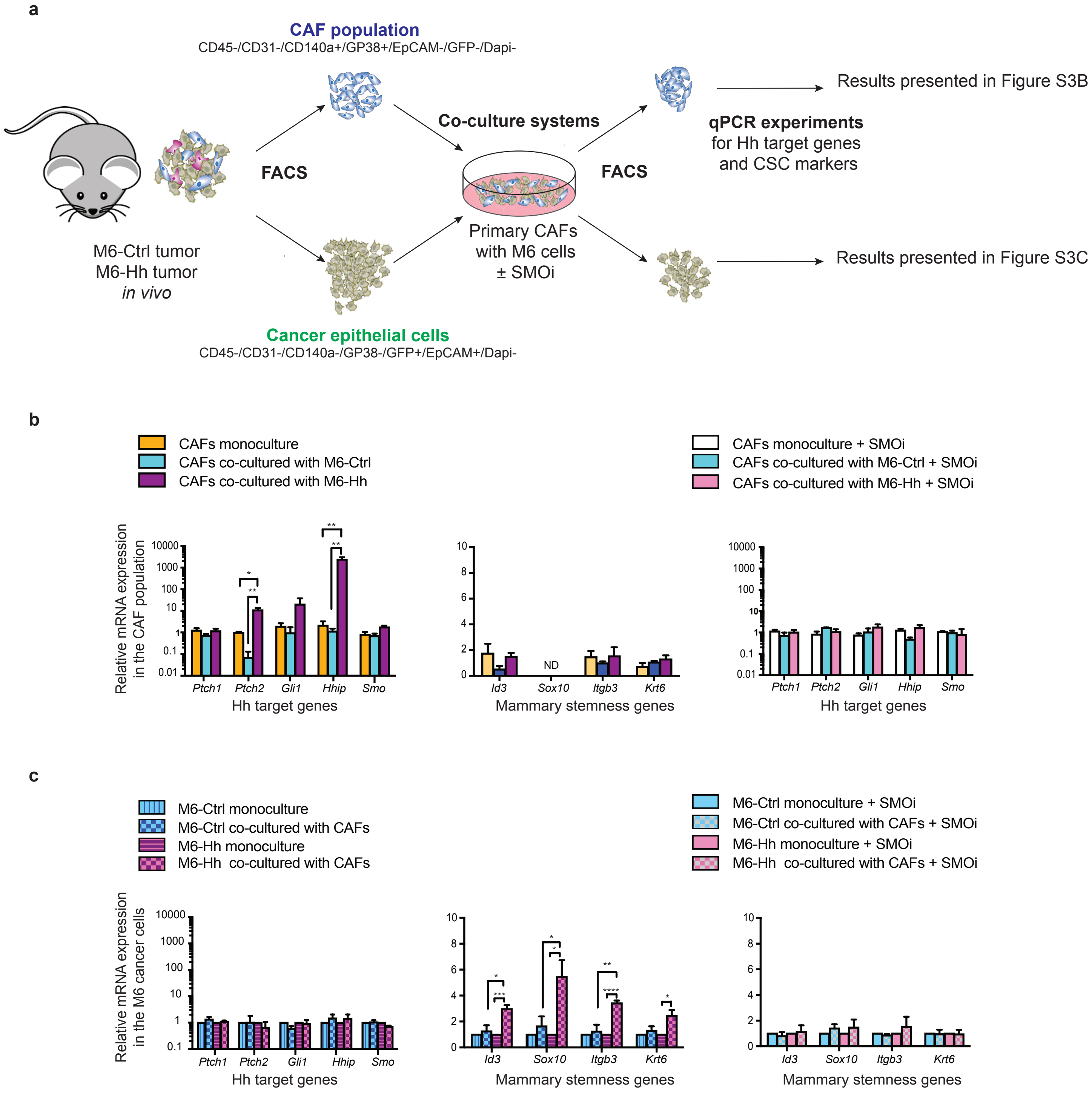

Supplementary Figure 3. Hh-activated CAFs drive a reversible stem-like phenotype in TNBC that can be reproduced using primary cells in vitro

(a) Scheme depicting the co-culture systems using primary CAFs and neoplastic cells from the same M6-Ctrl or M6-Hh murine tumor models. **(b)** Real time PCR measurement of Hh target genes and mammary CSC markers in primary CAFs co-cultured with M6 cells isolated from M6-Ctrl or M6-Hh primary tumors ± SMOi (10 µM). Canonical Hh target genes *Ptch2*, *Gli1* and *Hhip* are upregulated in the CAF population. **(c)** In turn, Hh-activated CAFs induce the expression of the stem cell markers *Id3*, *Sox10*, *Itgb3* and *Krt6* in the epithelial M6 cancer cells. Treatment with a SMOi inhibits paracrine Hh signaling pathway activation and reverses the stem-like phenotype to baseline (n = 4 biological replicates per group). Bars represent mean ± s.e.m; statistical significance was determined using unpaired two-tailed Student's t-test with equal s.d; * $P < 0.05$; ** $P < 0.01$; *** $P < 0.001$; **** $P < 0.0001$.

a

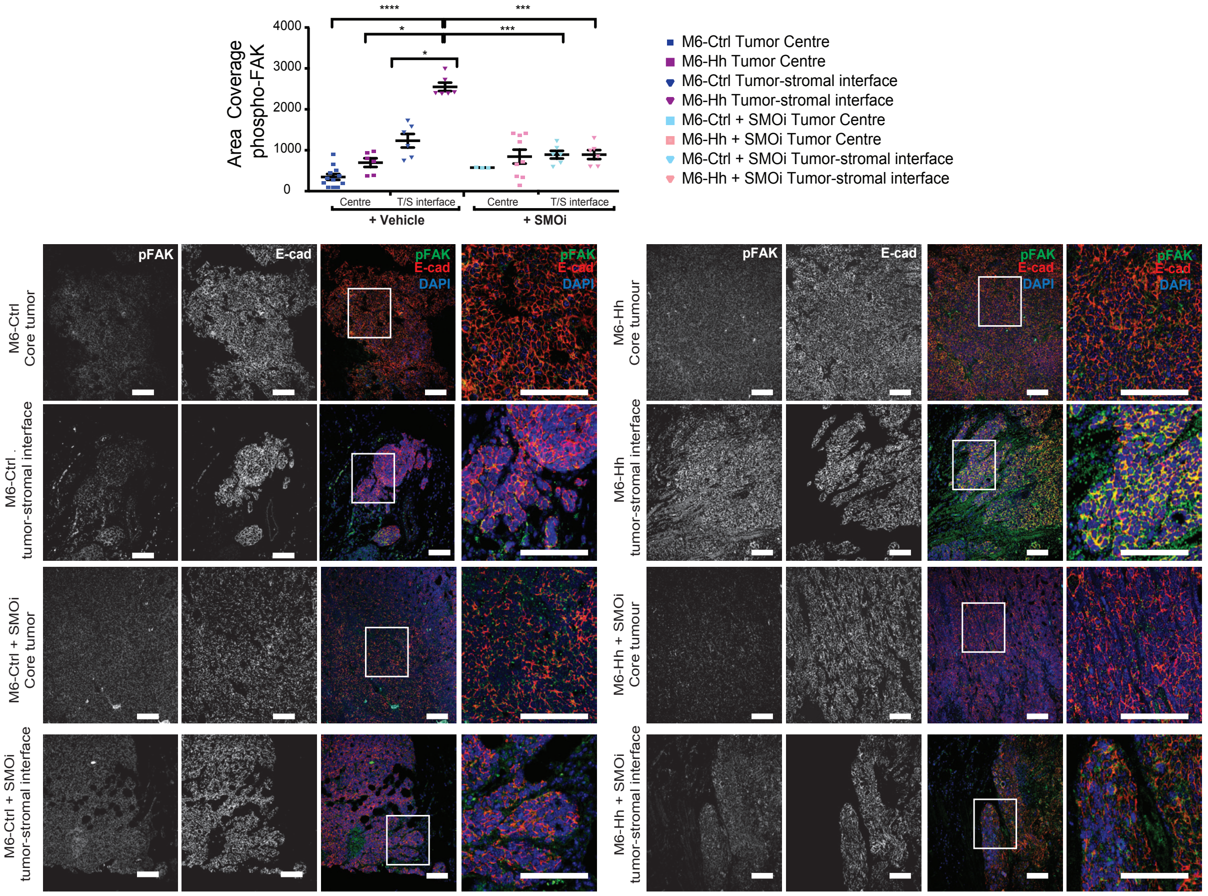
Area Coverage phospho-FAK
4000
3000
2000
1000
0
****
***
*
***
*
Centre
T/S interface
Centre
T/S interface
+ Vehicle
+ SMOi
M6-Ctrl Tumor Centre
M6-Hh Tumor Centre
M6-Ctrl Tumor-stromal interface
M6-Hh Tumor-stromal interface
M6-Ctrl + SMOi Tumor Centre
M6-Hh + SMOi Tumor Centre
M6-Ctrl + SMOi Tumor-stromal interface
M6-Hh + SMOi Tumor-stromal interface
pFAK
E-cad
pFAK
E-cad
DAPI
M6-Ctrl
Core tumor
M6-Ctrl
tumor-stromal interface
M6-Ctrl + SMOi
Core tumor
M6-Ctrl + SMOi
tumor-stromal interface
M6-Hh
Core tumour
M6-Hh
tumor-stromal interface
M6-Hh + SMOi
Core tumour
M6-Hh + SMOi
tumor-stromal interface

b

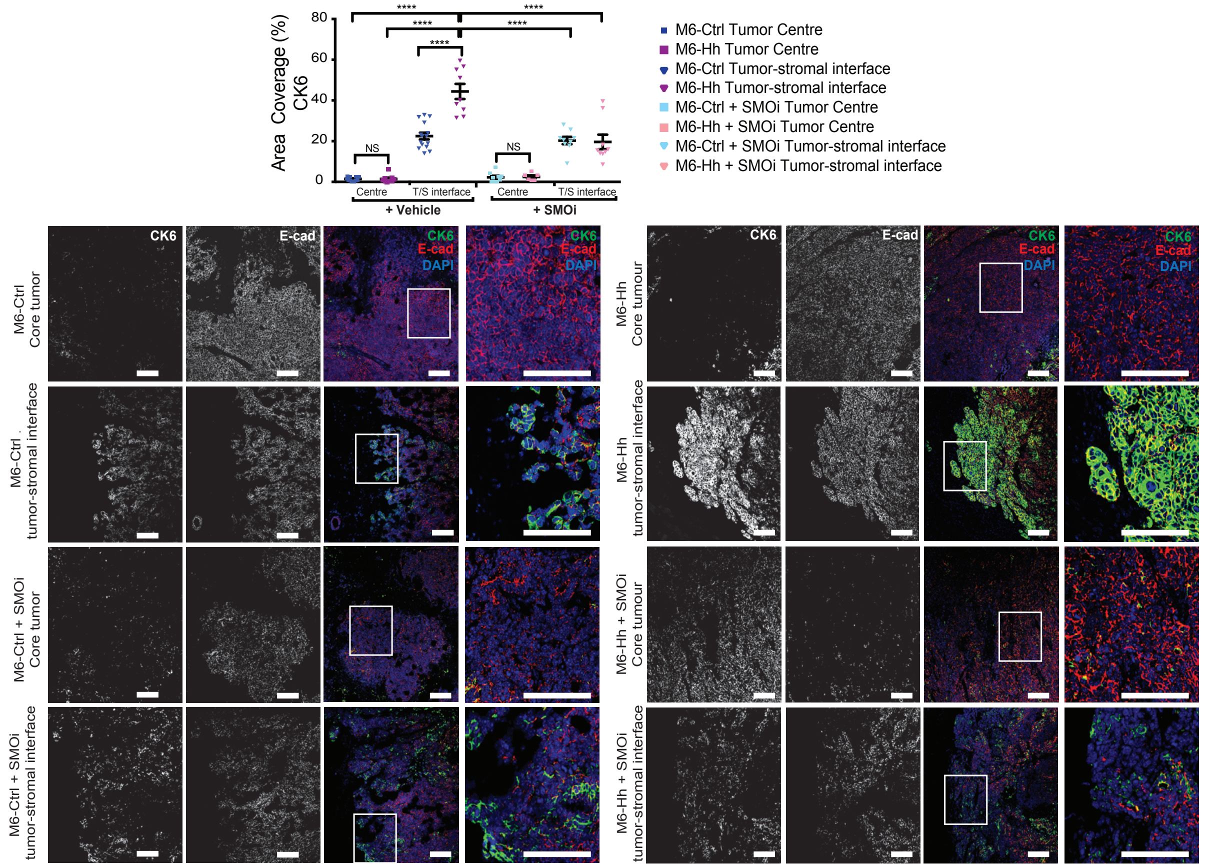
Area Coverage (%) CK6
80
60
40
20
0
****
****
****
****
****
NS
NS
Centre
T/S interface
Centre
T/S interface
+ Vehicle
+ SMOi
M6-Ctrl Tumor Centre
M6-Hh Tumor Centre
M6-Ctrl Tumor-stromal interface
M6-Hh Tumor-stromal interface
M6-Ctrl + SMOi Tumor Centre
M6-Hh + SMOi Tumor Centre
M6-Ctrl + SMOi Tumor-stromal interface
M6-Hh + SMOi Tumor-stromal interface
CK6
E-cad
CK6
E-cad
DAPI
M6-Ctrl
Core tumor
M6-Ctrl
tumor-stromal interface
M6-Ctrl + SMOi
Core tumor
M6-Ctrl + SMOi
tumor-stromal interface
M6-Hh
Core tumour
M6-Hh
tumor-stromal interface
M6-Hh + SMOi
Core tumour
M6-Hh + SMOi
tumor-stromal interface

Supplementary Figure 4. Enhanced mechanosignaling and breast cancer stemness upon Hh pathway activation occurs specifically at the tumor-stromal interface of Hh-expressing tumors

(a) Representative immunofluorescence images and quantification of phospho-FAK and **(b)** the mammary progenitor CK6 expression in M6-Ctrl and M6-Hh tumor specimens ± SMOi (100 mg/kg/bid). Expression was analyzed in dense cellular regions (core tumor) and at the tumor-CAF interface (enriched for collagen deposition). E-Cadherin was used to stain the cancer epithelium. Scale bars, 100 µm. Line at mean ± s.e.m for each treatment group; $n = 3$ biological replicates. Statistical significance was determined using Kruskal-Wallis test; * $P < 0.05$; *** $P < 0.001$; **** $P < 0.0001$.

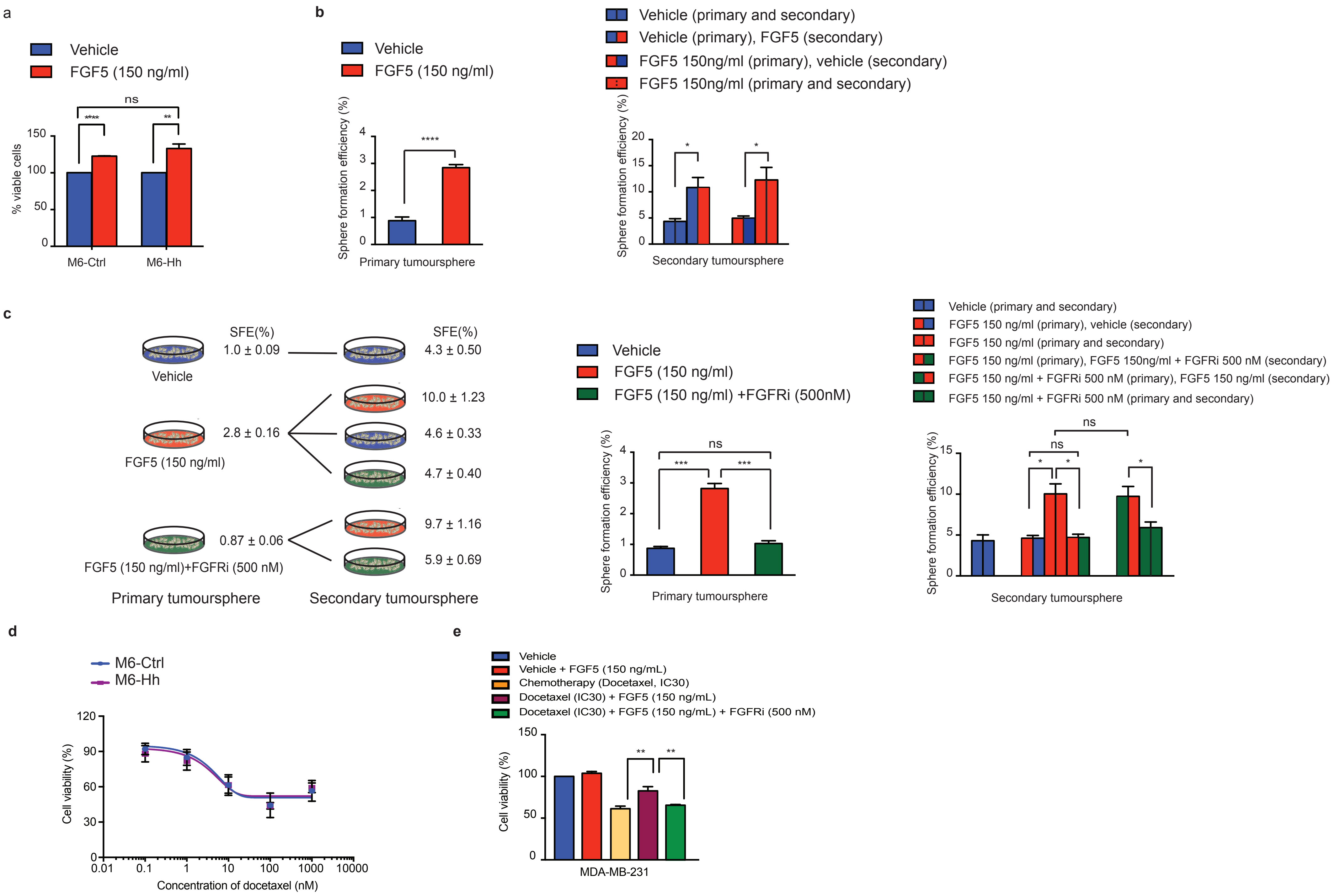

a
Vehicle
FGF5 (150 ng/ml)
ns
****
**
% viable cells
M6-Ctrl
M6-Hh
b
Vehicle
FGF5 (150 ng/ml)
Sphere formation efficiency (%)
****
Primary tumoursphere
Vehicle (primary and secondary)
Vehicle (primary), FGF5 (secondary)
FGF5 150ng/ml (primary), vehicle (secondary)
FGF5 150ng/ml (primary and secondary)
Secondary tumoursphere
c
SFE(%)
1.0 ± 0.09
Vehicle
SFE(%)
4.3 ± 0.50
10.0 ± 1.23
2.8 ± 0.16
4.6 ± 0.33
FGF5 (150 ng/ml)
4.7 ± 0.40
9.7 ± 1.16
0.87 ± 0.06
5.9 ± 0.69
FGF5 (150 ng/ml)+FGFRi (500 nM)
Primary tumoursphere
Secondary tumoursphere
Vehicle
FGF5 (150 ng/ml)
FGF5 (150 ng/ml) +FGFRi (500nM)
Vehicle (primary and secondary)
FGF5 150 ng/ml (primary), vehicle (secondary)
FGF5 150 ng/ml (primary and secondary)
FGF5 150 ng/ml (primary), FGF5 150ng/ml + FGFRi 500 nM (secondary)
FGF5 150 ng/ml + FGFRi 500 nM (primary), FGF5 150 ng/ml (secondary)
FGF5 150 ng/ml + FGFRi 500 nM (primary and secondary)
d
M6-Ctrl
M6-Hh
Cell viability (%)
Concentration of docetaxel (nM)
e
Vehicle
Vehicle + FGF5 (150 ng/mL)
Chemotherapy (Docetaxel, IC30)
Docetaxel (IC30) + FGF5 (150 ng/mL)
Docetaxel (IC30) + FGF5 (150 ng/mL) + FGFRi (500 nM)
Cell viability (%)
MDA-MB-231

Supplementary Figure 5. Recombinant FGF5 treatment modestly improves cell viability but facilitates the acquisition of a chemo-resistant niche enriched for CSC properties

(a) Cell viability of M6-Ctrl and M6-Hh cells treated with recombinant FGF5 under serum-free culture condition (*n* = 3 biological replicates with 6 technical replicates each). Significance was calculated using unpaired two-tailed Student's t-test; ** $P < 0.01$; **** $P < 0.0001$. **(b)** Sphere formation efficiency of M6-Ctrl primary and secondary tumorspheres treated with vehicle or recombinant FGF5 (*n* = 3 biological replicates with 3 technical replicates each). Statistical significance was determined by unpaired two-tailed Student's t-test with equal s.d; * $P < 0.05$, **** $P < 0.0001$. **(c)** Schema depicting primary and secondary tumorsphere assays using M6-Ctrl cells treated with vehicle (DMSO; blue), recombinant FGF5 (red) or with the FGFR inhibitor, NVP-BGJ398 (green) and associated sphere formation efficiency (SFE). Data represent mean ± s.e.m; sphere formation efficiency of primary and secondary tumorspheres (*n* = 3 biological replicates with 3 technical replicates each). Statistical significance was determined by unpaired two-tailed Student's t-test with equal s.d; * $P < 0.05$; *** $P < 0.001$. **(d)** *In vitro* sensitivity of M6-Ctrl (blue) and M6-Hh (magenta) cell lines to half-log serial dilution of docetaxel chemotherapy. Single-agent response curves were generated using the cell viability alamarblue® reduction assay (*n = 5* biological replicates with 6 technical replicates each). **(e)** Cell viability with the human MDA-MB-231 cell line treated with indicated agents (*n* = 4 biological replicates with 6 technical replicates each). Statistical significance was determined using unpaired two-tailed Student's t-test with equal s.d; ** $P < 0.01$. Bars are presented as mean ± s.e.m

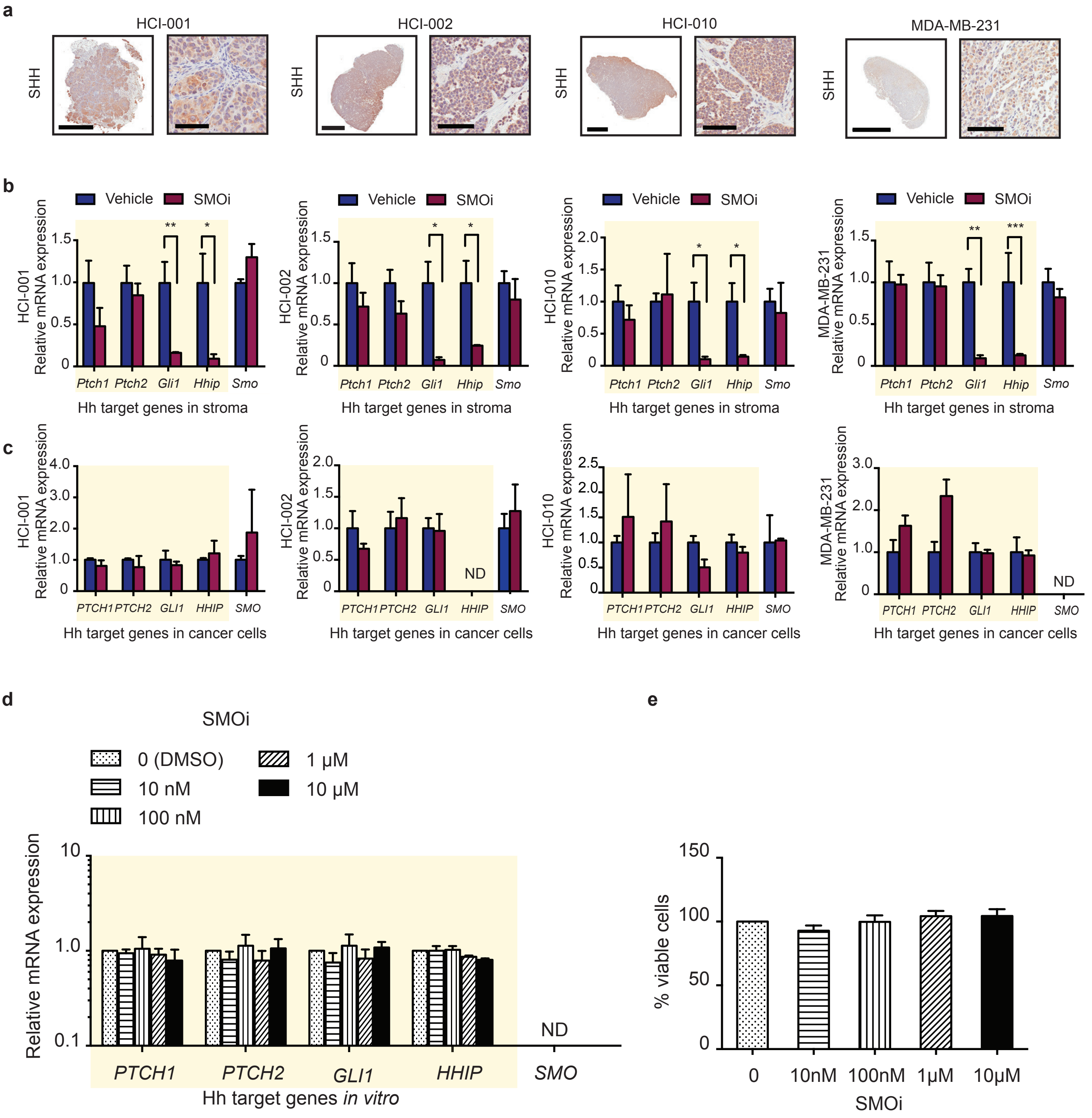

Supplementary Figure 6. Paracrine Hh signaling in human models of TNBC

(a) Representative SHH immunostaining of TNBC PDXs HCI-001, HCI-002, HCI-010 and the MDA-MB-231 xenograft model. Scale bars: (left panel), 4 mm; (right panel), 100 µm. **(b)** Real time PCR measurement of stromal Hh target genes in TNBC PDX and MDA-MB-231 models treated with vehicle or the SMOi, NVP-LDE225 (80 mg/kg/day; $n = 3$ biological replicates per treatment group). Statistical significance was determined using unpaired two-tailed Student's t-test with equal s.d; * $P < 0.05$; ** $P < 0.01$, *** $P < 0.001$. **(c)** Real time PCR measurement of Hh target genes in the corresponding tumor epithelial component of TNBC PDX and MDA-MB-231 models treated with vehicle or SMOi. **(d)** Lack of autocrine Hh signaling in the human MDA-MB-231 model of TNBC. Real time PCR measurement of Hh target genes in MDA-MB-231 cells treated with vehicle or SMOi ($n = 3$ biological replicates with 3 technical replicates each). **(e)** Cell viability of MDA-MB-231 cells treated with increasing doses of SMOi ($n = 3$ biological replicates with 6 technical replicates per treatment group). Bars represent mean ± s.e.m.

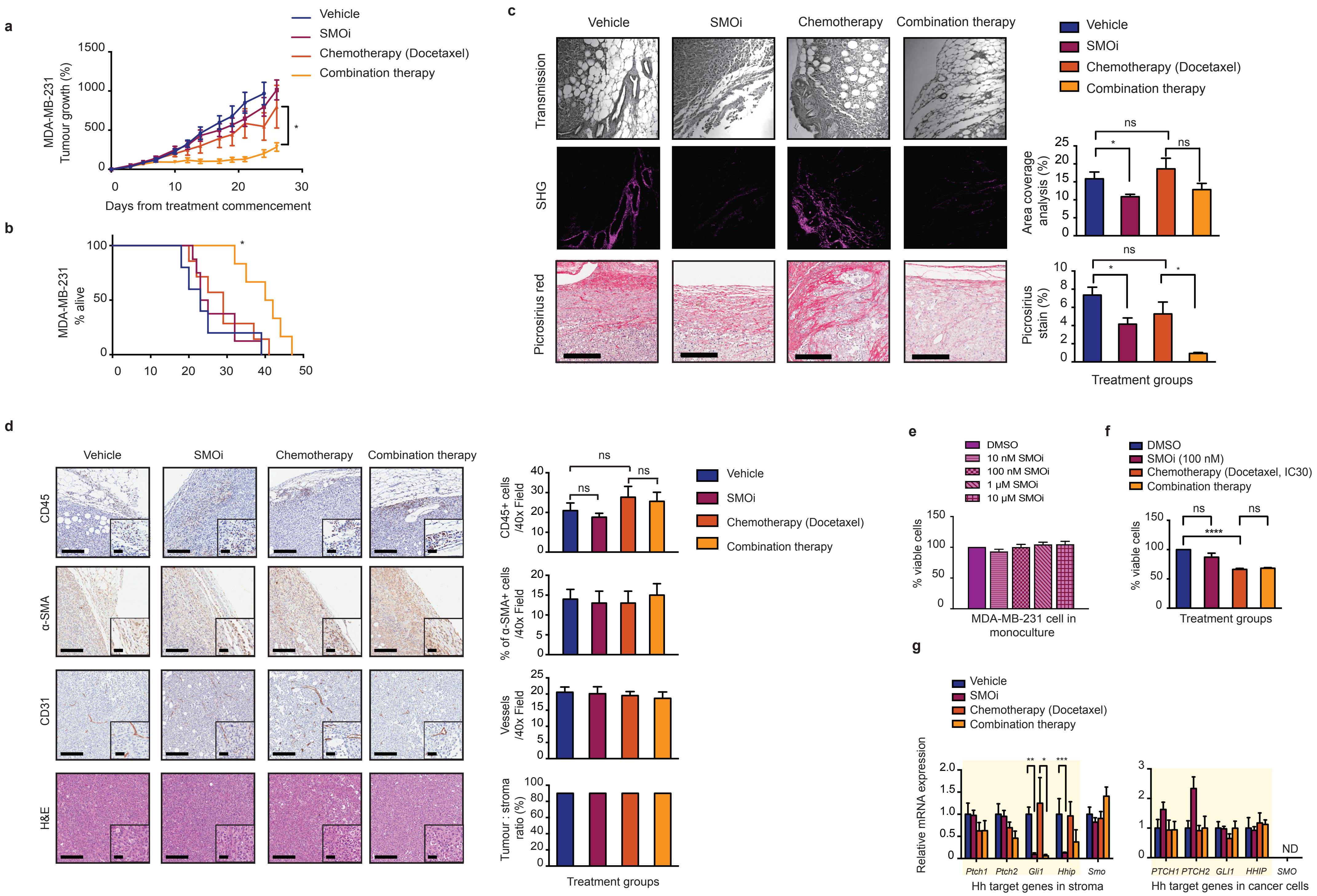

a
Vehicle
SMOi
Chemotherapy (Docetaxel)
Combination therapy
MDA-MB-231
Tumour growth (%)
Days from treatment commencement
b
MDA-MB-231
% alive
c
Vehicle
SMOi
Chemotherapy
Combination therapy
Transmission
SHG
Picrosirius red
Area coverage analysis (%)
Picrosirius stain (%)
Treatment groups
ns
d
CD45
α-SMA
CD31
H&E
CD45+ cells /40x Field
% of α-SMA+ cells /40x Field
Vessels /40x Field
Tumour : stroma ratio (%)
Treatment groups
e
DMSO
10 nM SMOi
100 nM SMOi
1 μM SMOi
10 μM SMOi
% viable cells
MDA-MB-231 cell in monoculture
f
SMOi (100 nM)
Chemotherapy (Docetaxel, IC30)
g
Relative mRNA expression
Ptch1
Ptch2
Gli1
Hhip
Smo
Hh target genes in stroma
PTCH1
PTCH2
GLI1
HHIP
SMO
ND
Hh target genes in cancer cells

Supplementary Figure 7. Therapeutic synergy of combination treatment in the MDA-MB-231 tumor model

(a-d) MDA-MB-231 tumors were treated with vehicle (blue), SMOi (NVP-LDE225; 80 mg/kg/day; magenta), chemotherapy (docetaxel; dark orange) or NVP-LDE225 + docetaxel (orange line). n = 7 mice per treatment group. **(a)** Tumor growth curves. Statistical significance was determined using unpaired Student's t test between combination therapy and docetaxel monotherapy; * $P < 0.05$. **(b)** Kaplan-Meier curves of mice overall survival of each treatment group. Statistical significance was determined using the Log-rank test of LDE225 + docetaxel versus docetaxel; * $P < 0.05$. **(c)** Representative images and quantification of SHG and picrosirius red staining. Scale bars; 200 µm. Statistical significance was determined using unpaired two-tailed Student's t-test with equal s.d; * $P < 0.05$. **(d)** Representative images of immunostaining for immune cells (CD45), CAFs (α-SMA), endothelial cells (CD31) and representative hematoxylin and eosin images of tumor:stroma ratio. Scale bars: 200 µm (main panels) and 100 µm (insets). Quantitative analysis of immune cells, CAFs, endothelial cells and tumor:stroma ratio. n = 6-8 biological specimens per treatment group. **(e)** Cell viability of MDA-MB-231 cells treated with increasing doses of SMOi *in vitro*. n = 3 biological replicates with 6 technical replicates per treatment group. **(f)** Cell viability of MDA-MB-231 cells treated *in vitro* with DMSO (blue), SMOi (NVP-LDE225, 100 nM; magenta), chemotherapy (Docetaxel, IC_{30}; dark orange) and combination therapy with SMOi and chemotherapy (orange; n = 3 biological replicates with 6 technical replicates each). Significance was calculated using unpaired two-tailed Student's t-test; **** $P < 0.0001$. **(g)** Real time PCR measurement of murine stromal and human epithelial Hh target genes in MDA-MB-231 tumors *in vivo* treated with vehicle, SMOi (NVP-LDE225; 80 mg/kg/day), chemotherapy (docetaxel; 15 mg/kg/week) and combination therapy with SMOi (80 mg/kg/day) and chemotherapy (15 mg/kg/week; n = 5 biological replicates per treatment group). Statistical significance was determined using unpaired two-tailed Student's t-test with equal s.d; * $P < 0.05$; ** $P < 0.01$; *** $P < 0.001$. Bars represent mean ± s.e.m.

Supplementary Table 1: Clinico-pathological characteristics of patients enrolled in the EDALINE Phase 1 clinical trial

ID	Dose Level	Age	Menopausal status	ECOG Performance Status	Number of prior lines for metastatic disease	Metastatic locations	Visceral involve-ment	Time to progression (days)	Best overall tumor response	Histological grade	Ki67 Index (%)	SHH H score	GLI Stroma Intensity	Pathway Activation
115	DL1	36,50	Premenopausal	1	0	Lymph node<br>Bone	No	31	PD	G3	NA	270	1	No
142	DL1	48,03	Postmenopausal	1	3	Lung<br>Lymph node<br>Bone<br>Brain	Yes	29	PD	G3	65	100	1	No
124	DL1	64,67	Postmenopausal	1	0	Lymph node<br>Breast	No	42	PD	G3	75	120	1	No
134	DL1	54,54	Postmenopausal	0	0	Lymph node<br>Soft tissue<br>Breast<br>Bone	No	42	PD	G3	90	160	1	No
139	DL1	26,76	Premenopausal	0	1	Lung<br>Liver<br>Lymph node<br>Bone	Yes	84	PD	G2	70	100	1	No
**101**	**DL2**	**35,53**	**Premenopausal**	**0**	**1**	**Skin**	**No**	**43**	**PD**	**G3**	**90**	**240**	**2**	**Yes**
110	DL2	46,02	Premenopausal	1	0	Lymph node	No	64	PD	G3	65	80	N/A	No
161	DL2	76,09	Postmenopausal	1	2	Breast<br>Bone	No	20	PD	G3	20	140	1	No
131	DL2	44,19	Premenopausal	0	0	Skin	No	42	PD	G3	40	100	1	No
**155**	**DL3**	**54,09**	**Postmenopausal**	**0**	**0**	**Lung**	**Yes**	**203**	**CR**	**Gx**	**75**	**200**	**2**	**Yes**
**154**	**DL3**	**57,36**	**Postmenopausal**	**0**	**0**	**Liver**<br>**Lung**	**Yes**	**155**	**SD**	**G3**	**30**	**180**	**2**	**Yes**
**195**	**DL3**	**51,5**	**Postmenopausal**	**0**	**0**	**Lung**	**Yes**	**188**	**SD**	**G2**	**35**	**N/A**	**N/A**	**N/A**

PD: progressive disease; CR: complete response; SD: stable disease.

Supplementary Table 2: Treatment characteristics of patients enrolled in the EDALINE Phase 1 clinical trial

Screening number	Patient ID	Dose Level	Number of cycles	Duration of treatment (days)	Best tumor response according to RECIST version 1.1
EDALINE_ESP0013001	115	Docetaxel 75mg/m^2 + LDE225 400mg PO daily	2	31	PD
EDALINE_ESP0082001	142	Docetaxel 75mg/m^2 + LDE225 400mg PO daily	2	29	PD
EDALINE_ESP0082002	124	Docetaxel 75mg/m^2 + LDE225 400mg PO daily	2	42	PD
EDALINE_ESP0082003	134	Docetaxel 75mg/m^2 + LDE225 400mg PO daily	2	42	PD
EDALINE_ESP0110001	139	Docetaxel 75mg/m^2 + LDE225 400mg PO daily	4	84	PD
EDALINE_ESP0003001	101	Docetaxel 75mg/m^2 + LDE225 600mg PO daily	2	43	PD
EDALINE_ESP0044001	110	Docetaxel 75mg/m^2 + LDE225 600mg PO daily	2	42	PD
EDALINE_ESP0082004	161	Docetaxel 75mg/m^2 + LDE225 600mg PO daily	3	64	PD
EDALINE_ESP0082005	131	Docetaxel 75mg/m^2 + LDE225 600mg PO daily	1	20	PD
EDALINE_ESP0003002	155	Docetaxel 75mg/m^2 + LDE225 800mg PO daily	8	168	CR
EDALINE_ESP0082006	154	Docetaxel 75mg/m^2 + LDE225 800mg PO daily	6	155	SD
EDALINE_ESP0110003	195	Docetaxel 75mg/m^2 + LDE225 800mg PO daily	9	188	SD

PD: progressive disease; CR: complete response; SD: stable disease.

Supplementary Table 3 – List of antibodies

Antibody	Manufacturer (Clone) and Catalogue number	Application and Dilution	Antigen Retrieval
Rabbit polyclonal anti-SHH	Santa Cruz Biotechnology (H160) Catalogue number: sc-9024	IHC, 1:40 (PDX)	PDX tissue: 40 min in boiling waterbath (DAKO pH 9 solution; s2367)
		IHC, 1:80 (mouse)	Mouse tissue: 20 min in boiling waterbath (DAKO pH9 solution; s2367)
Rabbit polyclonal anti-GLI1	Santa Cruz Biotechnology (H300) Catalogue number: sc-20687	IHC, 1:50	30s at maximum temperature and pressure in a pressure cooker in DAKO pH 6.1 solution (s1699)
Rabbit polyclonal anti-mouse alpha-SMA	Abcam Catalogue number: ab5694	IHC, 1:100	HIER 30 min with ER2 at 100°C
Rat anti-mouse CD31	Dianova (SZ31) Catalogue Number: DIA-310	IHC, 1:100	30s at maximum temperature and pressure in a pressure cooker in DAKO pH 6.1 solution (s1699)
Biotin Rat anti-mouse CD45	BD Pharmigen (30-F11) Catalogue number 553077	IHC, 1:200	20 min in boiling waterbath (DAKO pH 6.1 solution; s1699)
Phospho-Histone H3 (Ser10)	Cell Signalling Catalogue number #9701	IHC, 1:100	PDX tissue: HIER 30 min with ER2 at 100°C
			Mouse tissue: HIER 30 min with ER1 at 100°C
Rabbit anti-Phospho-FGF Receptor (Tyr 653/654)	Cell signalling Catalogue number: #3471	IHC, 1:50	Mouse tissue: HIER 30 min with ER2 at 100°C
Purified mouse anti-human ALDH1	BD Tansduction Laboratories (44/ALDH) Catalogue number: 611194	IHC, 1:200<br>IF, 1:50	PDX tissue: HIER 30 min with ER2 at 100°C
Anti-mouse Keratin 6 polyclonal antibody	Covance Catalogue number: PRB-169P	IHC, 1:400<br>IF, 1:100	IHC: HIER 10 min with Enzyme 2 (Bond Enzyme Pretreatment Kit, Leica Biosystems) at 37°C<br>IF: 12 min in a pressure cooker in 10 mMol/L Citrate (pH 6.0)
Anti-E-cadherin	BD Biosciences Catalogue number : 36/E-Cadherin	IF, 1:100	12 min in a pressure cooker in 10 mMol/L Citrate (pH 6.0)
Anti-p(Tyr397)-FAK	Invitrogen Catalogue number : 141-9	IF, 1:50	12 min in a pressure cooker in 10 mMol/L Tris-Cl (pH 9.0)

AlexaFluor secondary antibodies:<br>Goat anti-rabbit AlexaFluor 488/594 or goat anti-mouse AlexaFluor 488/594	Invitrogen	IF: 1:500	NA

Antibody	Manufacturer (Clone) and Catalogue number	Application and Dilution
PerCp/Cy5.5 anti-mouse EpCAM CD326	BD Biosciences (clone G8.8); Catalogue Number: 118220	FACS, 1:500
Anti-mouse CD45 APC-eFluor780	eBioscience (clone 30-F11); Catalogue number: 47-0451-82	FACS, 1:500
APC anti-mouse CD140a	Biolegend (clone APA5); Catalogue number: 135908	FACS, 1:100
PE anti-mouse Podoplanin	Biolegend (clone 8.1.1); Catalogue number: 127408	FACS, 1:1000
Biotin Rat anti-mouse CD31 (PECAM)	BD Pharmingen (clone 390); Catalogue number: 558737	FACS, 1:40
Biotin Rat anti-mouse TER-119	BD Pharmingen; Catalogue number: 553672	FACS, 1:80
Biotin Rat anti-mouse CD45	BD Pharmingen (30-F11); Catalogue number: 553078	FACS, 1:100
Biotin anti-mouse BP-1	eBioscience (6C3); Catalogue number: 13-5891-82	FACS, 1:50
PE Rat anti-mouse CD24	BD Biosciences (M1/69); Catalogue number: 553262	FACS, 1:400
APC/Cy7 anti-mouse CD29	Biolegend (HMB1-1); Catalogue number: 102226	FACS, 1:100
APC anti-mouse CD61	Invitrogen; Catalogue number: MCD6105	FACS, 1:50
Purified rat Anti-Mouse CD16/CD32 (Mouse BD Fc Block)	BD Biosciences; Catalogue number: 553141	FACS, 1:200
Streptavidin APC-Cy™7	BD Biosciences; Catalogue number: 554063	FACS, 1:400
Brilliant Violet 421™ Streptavidin	Biolegend; Catalogue number: 405226	FACS, 1:400

Supplementary Table 4: List of probes and qRT-PCR programs used

Gene	Roche Universal Probe Library System		
	**Forward Primer**	**Reverse Primer**	**UPL Probe**
Ptch1	GGC CTG GCA GAG GAC TTA C	GGA AGC ACC TTT TGA GTG GA	10
Ptch2	GTC CAC CTA GTG CTC CCA AC	CTC AGC TCC TGA GCC ACA TT	40
Smo	CCA CCC TGC TCA TCT GGA	CTT GGC GAT CAT CTT GCT CT	104
Gli1	GGA CCC ACT CCA ATG AGA AG	CAT GCA CTG TCT TCA CGT GTT	33
Hhip	GTG TTC GGA GAT CGC AAT G	TTT TCT TGC CAT TGC TTG GT	67
Fap	CGT GTA TCG AAA ACT GGG TGT	AAA CCC ATT TCT ATG AAT TTT CTG AC	100
Acta2	CTC TCT TCC AGC CAT CTT TCA T	TAT AGG TGG TTT CGT GGA TGC	58
Cd31	CGG TGT TCA GCG AGA TCC	ACT CGA CAG GAT GGA AAT CAC	45
Cd45	CGG GAT GAG ACA GTT GAT GA	GTA TTC TGC GCA CTT GTT CCT	88
Cd68	GAC CTA CAT CAG AGC CCG AGT	CGCCATGAATGTCCACTG	96
Krt6a/b	GGA AAT TGC CAC CTA CAG GA	GGT GGA CTG CAC CAC AGA G	12
β-actin	CTA AGG CCA ACC GTG AAA AG	ACC AGA GGC ATA CAG GGA CA	63
Gapdh	GGG TTC CTA TAA ATA CGG ACT GC	CCA TTT TGT CTA CGG GAC GA	52
Hprt	GGA GCG GTA GCA CCT CCT	AAC CTG GTT CAT CAT CGC TAA	69

	**TaqMan Gene Expression Assay**
SHH	Hs00179843_m1
PTCH1	Hs00181117_m1
PTCH2	Hs01085642_m1
SMO	Hs01090242_m1
GLI1	Hs01110766_m1
HHIP	Hs01011015_m1
GAPDH	Hs02758991_s1
Shh	Mm00436528_m1
Ptch1	Mm00436026_m1
Ptch2	Mm00436047_m1
Smo	Mm01162710_m1
Gli1	Mm00494654_m1
Hhip	Mm00469580_m1
Fgf5	Mm01722391_m1
Id3	Mm00492575_m1
Gpc3	Mm00516722_m1
Thy1	Mm00493681_m1

Sox10	Mm00493681_m1
Fap	Mm01329177_m1
Acta2	Mm01546133_m1
Pdgfra	Mm00440701_m1
Gp38	Mm00494716_m1
β-actin	Mm01205647_g1
Gapdh	Mm99999915_g1

Roche LightCycler480 Program

Target temp (°C)	Acquisition mode	Hold	Ramp rate (°C/s)	Sec Target (per °C)	Step size (°C)
**Pre-Incubation**					
94	None	7 min	4.8	0	0
**Amplification**					
94	None	15 sec	4.8	0	0
60	None	30 sec	2.5	50	0.5
72	Single	15 sec	1.5	0	0
**Cooling**					
40	None	30 sec	2.5	0	0

ABI Prism 7900HT Sequence Detection System Program

Step			Time	Temp (°C)
**UDG Incubation**	Hold		2 min	50
**AmpliTaq Gold, UP Enzyme Activation**	Hold		10 min	95
**PCR**	Cycle (40 cycles)	Denature	15 sec	95
		Anneal/Extend	1 min	60
