# Supplementary material for: Targeting stromal remodeling and cancer stem cell plasticity overcomes chemoresistance in triple negative breast cancer
Source: Nat Commun. 2018 Jul 24;9:2897. doi: 10.1038/s41467-018-05220-6 (PMC6057940; doi:10.1038/s41467-018-05220-6)
Supplement: Supplementary file 2 — Description of Additional Supplementary Files [file 41467_2018_5220_MOESM2_ESM.pdf]

Description of Additional Supplementary Files

File Name: Supplementary Data 1
Description: contains the list of differentially expressed genes in the bulk epithelial and stromal fractions of M6-Hh expressing tumors in comparison to M6-Ctrl or M6-Hh tumor + SMOi, as well as results of Gene Set Enrichment analysis.

File Name: Supplementary Data 2
Description: contains the list of differentially expressed genes in the CAF population of M6-Hh expressing tumors in comparison to CAFs from M6-Ctrl or M6-Hh tumor + SMOi from single cell RNA-Seq analysis.
